# High Potential Isoindoline‐Based Nitroxides Posolytes for Aqueous Organic Redox Flow Batteries

**DOI:** 10.1002/cssc.202502461

**Published:** 2026-02-01

**Authors:** Karim Boutamine, Gilles Casano, Patricia Bassil, Sébastien Gauden, Cecilia Poderi, Emilie Pepe, Frédéric Favier, Steven Le Vot, Olivier Ouari

**Affiliations:** ^1^ Aix Marseille Univ CNRS, ICR UMR 7273 Marseille France; ^2^ ICGM University Montpellier, CNRS, ENSCM Montpellier France; ^3^ Réseau sur le Stockage Electrochimique de l’Energie (RS2E) CNRS Amiens France

**Keywords:** [2 + 2 + 2] cyclotrimerization, aqueous organic redox flow battery, isoindoline, nitroxide, novel synthetic strategy, posolyte

## Abstract

The growing transition from fossil fuels to renewable energy sources such as wind and solar requires efficient stationary energy storage systems to ensure grid stability. Among emerging technologies, redox flow batteries (RFBs) offer a promising solution due to their unique decoupling of energy and power capacities, allowing flexible system design. Recent advances in organic RFBs (ORFBs) have highlighted redox‐active organic molecules as sustainable alternatives to conventional vanadium‐based systems, addressing issues of cost and corrosivity. In particular, nitroxide radicals such as tetramethylpiperidinyloxyl (TEMPO) derivatives have demonstrated high reversibility and fast kinetics in aqueous systems, though the stability of their oxidized N‐oxoammonium form remains a challenge for long‐term storage. Isoindoline‐based nitroxides offer potential for enhanced stability but have been limited by complex and low‐yield synthetic routes. Herein, we present a convenient metal‐catalyzed [2 + 2 + 2] intermolecular cycloaddition strategy for the synthesis of isoindoline‐based nitroxides and their aza analogs, including two new candidates, TC‐TMIO and PPO. Electrochemical characterization reveals that PPO, a cationic 2,3‐dihydropyrrolo[3,4‐c]pyridinium nitroxide, exhibits an oxidation potential 220 mV higher than the benchmark 4‐TMA‐TEMPO and achieves solubility exceeding 3 M in 1 M NaCl aqueous solution. Preliminary stability assessments of the PPO and RFB testing using a methyl viologen/PPO system demonstrate its potential as a high‐performance, sustainable posolyte for aqueous ORFBs.

## Graphical abstract

A convenient metal‐catalyzed [2 + 2 + 2] cycloaddition enables the synthesis of isoindoline‐based nitroxides as new posolytes for aqueous organic redox flow batteries. The cationic PPO exhibits a 220 mV higher oxidation potential than 4‐TMA‐TEMPO, 3 M solubility in 1 M NaCl, and a cell voltage of 1.56 V. Flow battery tests reveal PPO's promising performance for sustainable energy storage.

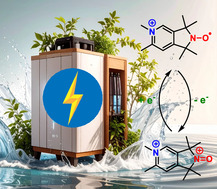

## 1 Introduction

**FIGURE 1:**
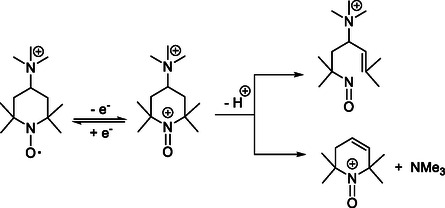
Structure of 4‐TMA‐TEMPO nitroxide radical and its one‐electron oxidation product (oxoammonium ion). Possible degradation pathways (ring opening and Hofmann elimination) induced by deprotonation of the oxoammonium ion.

Renewable power plants such as wind farms and photovoltaics are critical today to the shift from fossil fuels to renewable and sustainable energy sources. Stationary energy storage technologies are key partners to the widespread integration of these inherently intermittent, nondispatchable energy sources into the grid while ensuring grid stability. Redox flow battery (RFB) technology is emerging as a suitable and promising option, notably due to the decoupling of power output and energy. The amount of energy stored is determined by the size of the electrolyte tanks, while the power output is dictated by the size of the electrochemical stack. This decoupling enables RFBs to be scaled independently to meet specific energy and power requirements [1, 2, 3]. Thereby, RFBs are easily tailored to match the needs of a broad range of applications (off‐grid systems, backup power, and demand response batteries), in comparison with other technologies such as lithium‐ion batteries on the one hand and pumped storage hydropower on the other. Recent developments have shown that redox‐active organic molecules (ROM) can be valuable alternatives to counterbalance the limitations of commercially implemented vanadium RFB, notably their high and volatile cost, the use of corrosive acid‐based electrolytes and their limited energy densities [4, 5, 6, 7]. Organic RFBs are seen as a promising alternative due to their use of abundant and sustainable materials, but their performance and durability still require further improvement. The rational design of ROMs relies on chemical tailoring to improve key properties such as water solubility, redox potentials and kinetics, and stability to propose efficient, low‐cost, sustainable, and safe aqueous organic RFBs (AORFBs). In the quest for efficient organic posolytes for RFB, nitroxide radicals such as tetramethylpiperidinyloxyl (TEMPO) derivatives have recently attracted a lot of attention for AORFBs [8, 9, 10, 11, 12, 13, 14, 15]. The fast and reversible one‐electron reaction between the nitroxide and N‐oxoammonium couple (Figure 1), the suitable redox potentials and the rich chemistry to tailor the nitroxide molecules have opened promising perspectives. Although nitroxides are stable in aqueous solution in a wide range of pH values, their respective N‐oxoammonium cations appear to undergo slow decomposition reactions in aqueous solutions. Studies of the N‐oxoammonium fade in aqueous systems have proposed the involvement of the acidic methylene protons in the β‐position in an irreversible ring‐opening reaction [16, 17].

**SCHEME 1:**
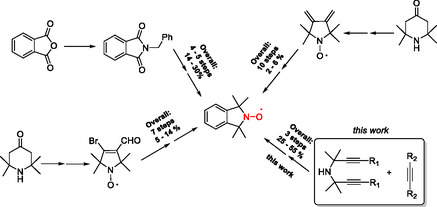
Reported synthetic routes for accessing isoindoline‐based nitroxides.

In 2016, Janoscka et al. introduced **4‐TMA‐TEMPO**, 4‐trimethylammonium‐TEMPO salt, as a promising posolyte for AORFB [18]. The higher and reversible oxidation potential of **4‐TMA‐TEMPO** respective to **4‐OH‐TEMPO** (0.99 V and 0.84 V vs. SHE, respectively), its fast redox kinetics, and its high solubility (2.3 M) in 1.5 M NaCl aqueous solution provided it as one of the most studied and promising posolytes for AORFB applications. Subsequently, it has been suggested by several studies that the trimethylammonium group plays a favorable role in the stability of the N‐oxoammonium cation, but the origin of this effect has not been identified. To our knowledge, limited examples of non‐TEMPO‐based nitroxides have been reported in the field of AORFBs, with only one example of isoindoline nitroxide in nonaqueous RFB [19]. The fused ring scaffold in isoindoline nitroxides have been reported to be less prone to ring‐opening degradation [20] and could provide opportunities to improve the stability of oxoammonium salts in aqueous systems. Despite their numerous advantages, widespread applications of isoindoline nitroxides may has been restricted by accessibility limitations (chemically and commercially). Three main synthetic approaches have been reported but they all present moderate to low‐yield schemes and usually restricted scope (Scheme 1). The tetraalkylation of N‐benzylphthalimide by excess of MeMgBr has been the most commonly used synthetic procedure [21, 22] to access 1,1,3,3‐tetramethylisoindolin‐2‐yloxyl nitroxide, and further functionalization approaches have been described in the last years [23, 24]. Hideg et al. have reported a strategy based on a Diels–Alder reaction involving a pyrrolidinedione and diethyl acetylenedicarboxylate to generate the isoindoline nitroxide frame after oxidative aromatization [25]. Nevertheless, the approach requires the six‐step synthesis of the diene precursor and sometimes the long heating period for the Diels–Alder reaction, which causes degradation or polymerization of the starting materials. Later, the same group reported an electrocyclic reaction of 3‐bromo‐4‐formyl‐2,2,5,5‐tetramethyl‐2,5‐dihydro‐1H‐pyrrol‐1‐yloxyl nitroxide to access isoindoline‐based nitroxides [26, 27, 28], but again, the synthetic pathway is long, with an overall yield inferior to 15% and exhibits limited structural functionalities.

Herein, we report a convenient and straightforward route for the preparation of isoindoline‐based nitroxides and their aza counterparts, such as 2,3‐dihydro‐1H‐pyrrolo[3,4‐c]pyridine derivatives, using a metal‐catalyzed [2 + 2 + 2] intermolecular cycloaddition approach. Two new nitroxides, **TC‐TMIO** and **PPO**, as candidate posolytes for AORFBs have been synthesized and their electrochemical properties, including the redox potential and kinetics, are reported. **PPO**, a cationic 2,3‐dihydropyrrolo[3,4‐c]pyridinium nitroxide, provides a redox potential surpassing the state‐of‐the‐art **4‐TMA‐TEMPO** nitroxide posolyte by 220 mV and exhibits a high solubility (>3 M) in 1 M NaCl aqueous solutions. Preliminary data on the stability of the **PPO** oxoammonium cation is also reported. The performance of a methyl viologen/**PPO** RFB is discussed and compared to the state‐of‐the‐art compound **4‐TMA‐TEMPO**.

## 2 Results and Discussion

### 2.1 Synthesis

The construction of functional fused rings using intermolecular cyclotrimerization reactions has attracted a growing interest in the last decade. This approach usually provides very efficient methods for the synthesis of complex products in a single step from simple building blocks, providing a high atom economy and broad reaction scope. Among them, the [2 + 2 + 2] cyclotrimerization of triple bonds are well‐established reactions for the synthesis of polysubstituted benzenes [29, 30, 31, 32, 33, 34]. In recent years, the use of transition metals such as Co, Fe, Ni, Rh, and Ru complexes has been reported to proceed cycloadditions under mild conditions using a broad range of unactivated nitriles and alkynes substituted at the α and β positions of substrates [35, 36, 37]. Therefore, the metal‐catalyzed intermolecular cycloaddition of alkynes and nitriles with α,ω‐dialkynylamines appears as an appealing method to access isoindoline and 2,3‐dihydropyrrolo[3,4‐c]pyridine nitroxides, respectively. As starting dialkynyl amines are readily accessible, we decided to further elaborate on the isoindoline and dihydropyrrolo[3,4‐c]pyridine synthesis using dialkyl acetylenedicarboxylate and acetonitrile substrates, respectively.

**SCHEME 2:**
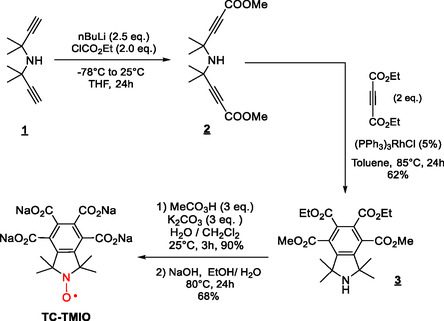
Synthesis of **TC‐TMIO**.

For **TC‐TMIO**, we selected the Rh‐catalyzed cyclotrimerization route, using Wilkinson's catalyst (5 mol%) and bis 4‐(methyl 4‐methylpent‐2‐ynoate)amine ( **2** ) with diethylacetylene carboxylate as the model reaction (Scheme 2).

**FIGURE 2:**
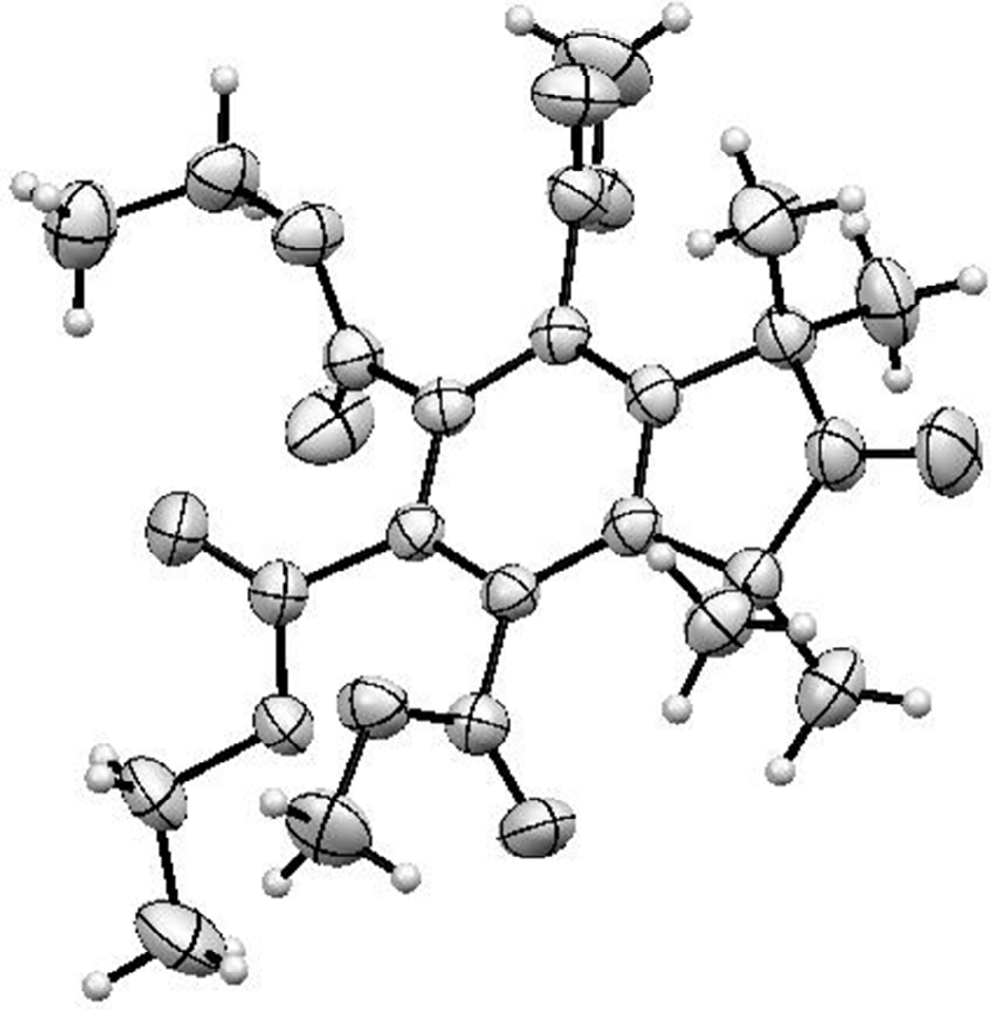
A view of the molecular structure of the nitroxide **TC‐TMIO**.

Isoindoline **3** was formed in 62% yield at 85°C in toluene with 5 mol% of catalyst and under an inert atmosphere, and these conditions enable a good conversion with limited side product formation. Oxidation of amine **3** by peracetic acid in a water/dichloromethane mixture at room temperature affords in 90% yield diethyl dimethyl 1,1,3,3‐tetramethylisoindoline‐N‐oxyl‐4,5,6,7‐tetracarboxylate (**TC‐TMIO**), which has not been reported yet to the best of our knowledge. The X‐ray crystal structure analysis confirmed the structure of nitroxide **TC‐TMIO** (Figure 2) and features a nearly planar isoindoline core with the tetracarboxylate groups pointing out of the plane.

**SCHEME 3:**
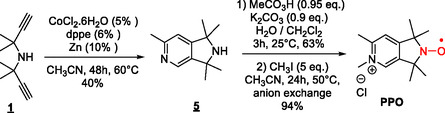
Synthesis of nitroxide **PPO**.

To document the applicability of the approach for the preparation of fused heterocycles, 2,3‐dihydropyrrolo[3,4‐c]pyridine was prepared using a Co‐catalyzed [2 + 2 + 2] cycloaddition, where the active catalyst was generated in situ from inexpensive and commercially available materials. The reaction of the bis(2‐methylbut‐3‐yn‐2‐yl)amine α,ω‐diynes ( **1** ) with acetonitrile in the presence of 5 CoCl_2_0.6 H_2_O (10%), dppe (12%), and Zn powder (20%) at 60°C yields the amine **5** in 40% yield (Scheme 3).

Selective oxidation of the secondary amine of **5** using peracetic acid at room temperature yields the corresponding nitroxide free radical in good yield (63%). Finally, the pyridine ring was further alkylated with methyl iodide to generate 1,1,3,3,5,6‐hexamethyl‐2,3‐dihydro‐1H‐pyrrolo[3,4‐c]pyridin‐5‐ium‐N‐oxyl nitroxide (**PPO**) in 94% yield.

### 2.2 Electrochemical Characterization of PPO and TC‐TMIO

The electrochemical properties of the newly reported **PPO** and **TC‐TMIO** nitroxides were characterized using cyclic voltammetry (CV) and rotating disk electrode (RDE) experiments in 1 M NaCl aqueous electrolytes. CV is a convenient method for preliminary assessing the reversibility and redox potential of the system.

Figure S18 shows a quasireversible redox potential (E^0^’) at 1.16 V (vs. SHE) of **PPO,** revealing a peak separation potential (ΔEp) of 70 mV that is almost independent of the scan rate, and with a cathodic to anodic peak current ratio (|Ipa/Ipc|) close to 1.

**FIGURE 3:**
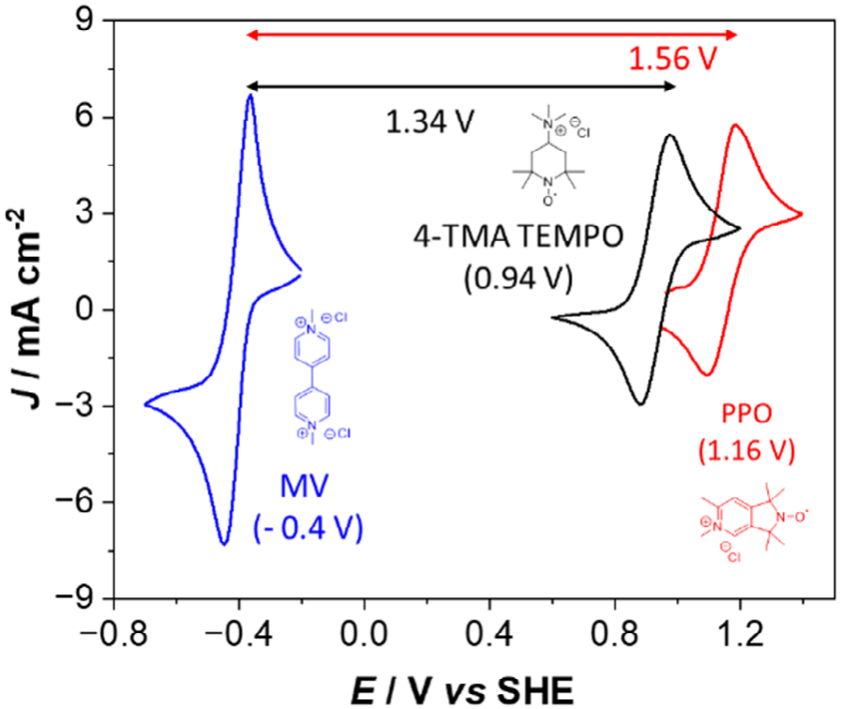
Cyclic voltammograms of 0.1 M **PPO** (red), **4‐TMA TEMPO** (black), and methyl viologen (blue) in 1 M NaCl (aq) solution. In parentheses the half‐wave potentials (E^0^’).

It is worth mentioning that **4‐TMA‐TEMPO**, one of the cationic posolytes of choice for today's AORFBs, shows a potential of 0.94 V (vs. SHE) (Figure S19), revealing an improved oxidation potential (+220 mV) for **PPO** (Figure 3) still suitable for the aqueous voltage window. Solubility of **PPO** reaches at least 3 M in 1 M NaCl aqueous electrolyte for both its reduced (uncharged) and oxidized (charged) states. These two properties are favorable criteria for AORFB full cells relative to reported nitroxide posolytes.

To determine the redox kinetic characteristics of **PPO**, further investigations were conducted using CV at various scan rates and RDE experiments at different rotation speeds. The apparent electron transfer rate constant (*k*_0,app_) and diffusion coefficient (*D*) were determined for **PPO**, **TC‐TMIO** and **4‐TMA‐TEMPO**, and the results are reported in Table 1 (additional details are provided in SI). For **PPO**, the *k*_0,app_ values were estimated to be 6.4 × 10^−3^ cm s^−1^ and 4.3 × 10^−3^ cm s^−1^ by CV (Figure S17) or RDE (Figure S20), respectively. The diffusion coefficients (*D*) of **PPO** have been determined as 2.3 × 10^−6^ cm^2^ s^−1^ and 2.9 × 10^−6^ cm^2^ s^−1^ from CV and RDE, respectively. Both kinetic characteristics are satisfying the targeted performances and are in line with values obtained for small‐sized nitroxides. Beyond the quantitative analysis, the values of *k*_0,app_ and *D* for **PPO** are very close to those of the state‐of‐the‐art **4‐TMA‐TEMPO** posolyte (Figures S19 and S22), indicating that, based on these preliminary electrochemical assessments, **PPO** is suitable for use in an RFB.

**TABLE 1:** Electrochemical Properties and diffusion coefficients for nitroxide posolytes.

	Redox potential (E^0^’) vs. SHE	Electron transfer rate (*k*_0_), 10^−3^ cm.s^−1^	Diffusion constant (*D*), 10^−6^ cm^2^.s^−1^	Solubility (M, in 1 M NaCl) solution
PPO	1.16^a^	6.4^a^ 4.3^b^	2.3^a^ 2.9^b^	3
4‐TMA‐TEMPO	0.94^a^	11.9^a^ 4.4^b^	3.3^a^ 2.8^b^	2.5
TC‐TMIO	0.91^a^	0.1^a^ nd	1.6^a^ nd	nd
TEMPO‐Sulfate [38]	0.81	1.91	2.98	nd

a Measured using CV experiments.

b Measured using rotating disk electrode experiments.

Values for sulfate TEMPO taken from reference [38].

**TC‐TMIO** shows a redox potential of 0.91 V (vs. SHE) (Figure S18). The electron transfer rate constant (*k*_0,app_) and diffusion coefficient (*D*) were found to be 0.1 × 10^−3^ cm s^−1^ and 1.6 × 10^−6^ cm^2^ s^−1^. Associated values for an anionic nitroxide posolyte (TEMPO‐sulfate) are indicated in Table 1 for comparison. Preliminary investigation of the cycling performances of **TC‐TMIO** for a long‐time experiment has been poor, mainly due to acid‐induced partial precipitation during cycling. For the rest of the study (battery cycling), only **PPO** and **4‐TMA‐TEMPO** are discussed.

### 2.3 Charge–Discharge Performance of PPO in AORBs

RFBs were assembled using **PPO** (0.1 M) and methyl viologen (**MV**) (1,1’‐bis(methyl)‐4,4’‐bipyridinium dichloride, 0.1 M) dissolved in 1.0 M NaCl as the posolyte and negolyte, respectively. A Fumasep FAA‐3‐50 (thickness 45 ‐ 55 µm, surface area 25 cm^2^) membrane was employed as an ion exchange membrane.

**FIGURE 4:**
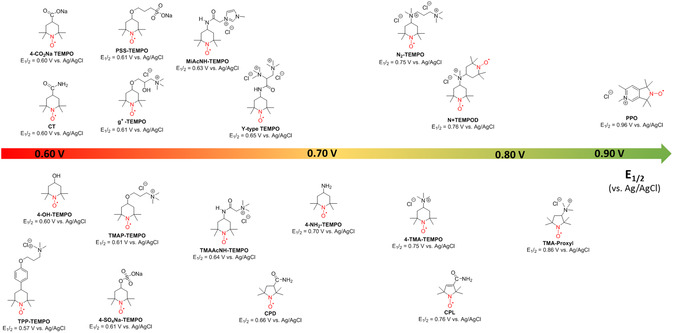
Redox potential and solubility of some nitroxide‐based posolytes reported for AORFB. 4‐COONa TEMPO [39], PSS‐TEMPO [40], MiAcNH‐TEMPO [41], N_2_‐TEMPO [13], CT [10], g^+^‐TEMPO [42], Y‐Type TEMPO [15], N + TEMPOD [11], 4‐OH‐TEMPO [43, 44, 45], TMAP‐TEMPO [8, 9, 12], TMAAcNH‐TEMPO [8, 9, 46], 4‐NH_2_‐TEMPO [44, 45, 47], 4‐TMA‐TEMPO [8, 9, 16, 18, 48, 49], TMA‐Proxyl [14], TPP‐TEMPO [9], 4‐S0_4_Na‐TEMPO [39], CPD [10], CPL [10].

**MV** was selected as a negolyte because of numerous studies using **MV** in AORFB reported in the literature and its commercial availability [5]. As shown in Figure 3, **PPO** resulted in a 16% improvement in cell voltage compared to the reference **4‐TMA‐TEMPO**. Moreover, the larger **PPO** solubility provides further improvements in energy density thanks to enhanced posolyte capacity. Considering a solubility of 3 M (1e^−^), the theoretical capacity of **PPO** is 80 Ah L^−1^, and the energy density of a battery combining **PPO** with **MV** (2.4 M, 1e^−^) as a negolyte is 57 Wh L^−1^, placing it as a promising organic posolyte when compared to data reported for nitroxides and highlighted in Figure 4.

**FIGURE 5:**
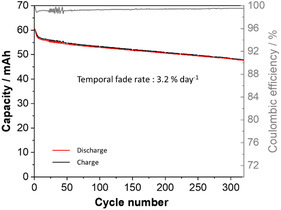
Charge/discharge capacity and coulombic efficiency as a function of the cycles number for **MV**/**PPO** AORFB. Conditions: anolyte: 75 ml of 0.1 M **MV** in 1 M NaClaq; catholyte: 25 ml of 0.1 M **PPO** in 1 M NaCl; Fumasep FAA‐3‐50 anion‐exchange membrane; current density 10 mA cm^−2^; temperature 23°C, time of cycling 6.8 days.

One of the key properties for long‐duration energy storage in AORBs is the stability of the system, notably the long‐term cycling. The charge–discharge experiment of **PPO‐**based RFB was conducted in the voltage range of 0.900–1.725 V at a current density of 10 mA cm^−2^ (Figure S23), and data were compared to **4‐TMA‐TEMPO‐**based AORFB (Figure S22). Detailed cycling protocols are provided in the SI. Since the posolyte is the focus of this study, the volume of the negolyte reservoir was selected to be three times larger than for the posolyte, maintaining the concentration identically, resulting in a configuration with a capacity‐limiting side (CLS) and a non‐CLS (NCLS). By using this unbalanced configuration, performances of the battery, including fade rate, can be solely attributed to the compartment of interest (CLS, in this case the posolyte) [50]. The cell voltage is 1.54 V, and the battery exhibits a low overpotential for charge and discharge, leading to good voltage efficiency. Using galvanostatic cycling at constant current followed by a potentiostatic regime at constant voltage [7, 51, 52], a discharge capacity of 60 mAh was obtained, recovering 90% of the theoretical capacity. As shown in Figure 5, the capacity then slightly decreases with time while maintaining excellent coulombic efficiency (>99%). For **4‐TMA‐TEMPO**, the first discharge led to a capacity of 62 mAh (Figure S23), with a subsequent decrease in capacity but to a lower extent compared to **PPO**.

From Figure 5 and S23, temporal capacity fade rates of 3.22% and 1.01% per day were found for **PPO** and **4‐TMA‐TEMPO**, respectively. The fade rate of **PPO** is in the range usually observed for other nitroxides, with **4‐TMA‐TEMPO** as one of the best standards. It is worth noting that most of the capacity loss occurs during the first cycles. Table S1 shows that from cycle 150 to cycle 200, the capacity fade rate is 1.9% and 0.70% per day for **PPO** and **4‐TMA‐TEMPO**, respectively.

In lab‐scale RFBs, the capacity fade rate has two main origins: the crossover of redox‐active molecules from one tank to the other and/or the chemical degradation of the redox species, which leads to capacity loss. In AORFBs, the latter phenomenon is usually the most critical, even if the degradation process is not understood. To identify the origin of observed capacity loss in both systems (**MV**–**PPO** and **MV**–**4‐TMA‐TEMPO**), CV, UV–visible spectroscopy, and ^1^H NMR spectroscopy analysis of electrolyte solutions (either posolytes or negolytes) have been performed before and after cycling (330 cycles, i.e., 6.8 days).

**FIGURE 6:**
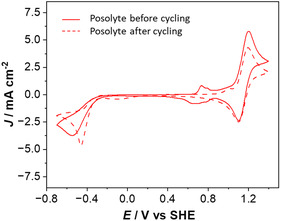
Cyclic voltammograms of **PPO** posolyte before (line) and after RFB 330 cycles (dash line).

CVs of the posolyte solution containing **PPO** (Figure 6), recorded over an extended potential range, show the response of the nitroxide/N‐oxoammonium redox couple at 1.16 V. Before battery cycling, during the negative scan, the reduction of the nitroxide moiety into hydroxylamine can be observed at −0.54 V. It can be noted also that a small redox system is also observed at around 0.7 V. This peak disappears quickly after a few cycles, likely due to the presence of residual iodine impurities originating from nonfully quantitative anion‐exchange metathesis. However, it disappears very quickly (as early as the second cycle) and does not interfere with the subsequent electrochemical analysis or affect the conclusions of the study. After cycling experiments, two comments are clear from the CV results. First, the current of the nitroxide/oxoammonium system has decreased, with an intensity loss (22.4%) matching closely the battery's capacity loss (21.9%). Second, oxidized **MV** is detected in the posolyte tank, indicating that **MV** passes through the membrane, confirming the nonnegligible crossover effect in a practical cell.

Figure S25 shows that, unlike **MV**, **PPO** nitroxide does not cross the membrane from the posolyte to the negolyte compartments. For **4‐TMA‐TEMPO**, a similar trend, for both electrolytes, is noted from CV, before and after cycling (Figure S10). However, the decrease in the current intensity corresponding to the nitroxide/oxoammonium couple is less pronounced (6.5%) for **4‐TMA‐TEMPO**, which is in good agreement with the battery's capacity fade (6.9%).

In recent years, it has been observed that the degradation of the TEMPO‐based posolyte stems mainly from the decay of the oxoammonium species (charged state) rather than the nitroxide species (discharged state). Numerous electrochemical studies have hypothesized possible chemical degradation mechanisms of the N‐oxoammonium species, mostly by a Hofmann‐like elimination and/or a ring‐opening reaction [16, 17]. Recently, Barbon et al. showed that the N‐oxoammonium cation at pH 9 reacts quickly with hydroxide ions to generate back the corresponding nitroxide alongside H_2_O and O_2_ in a 4/2/1 ratio, respectively [53]. This process can be seen as less detrimental than chemical alteration such as ring opening. The posolyte can thus be recovered in the discharged state, and the reaction corresponds to the self‐discharge of the battery.

**FIGURE 7:**
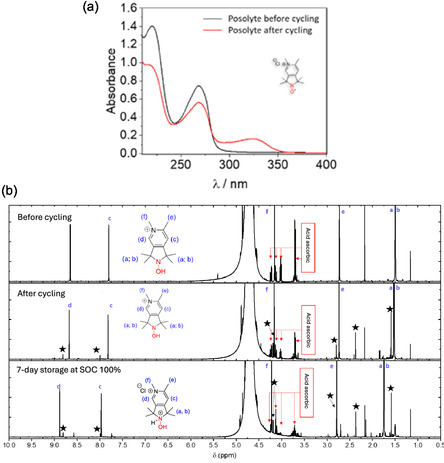
(a) UV–visible spectrum and (b) ^1^H NMR spectra of **PPO** electrolytes before and after cycling. Ascorbic acid has been added to the solution for the NMR experiments. Stars indicate the additional peaks observed after cycling.

Postmortem UV–visible and ^1^H NMR investigation of the **PPO** electrolyte solution revealed degradation products (Figure 7b). The appearance of a new chromophore at 324 nm and the decrease in the intensity of the two main bands at 220 and 268 nm indicate the transformation of the N‐oxoammonium (Figure 7a). Moreover, new peaks at 1.58, 1.83, 2.37, 7.99, and 8.81 ppm were observed by ^1^H NMR (Figure 7b).

To better understand **PPO** and **4‐TMA‐TEMPO** electrolyte degradation and the possible influence of cycling conditions and/or the presence of **MV** therein, two electrolytes prepared from the same solutions were charged to 100% SOC in two different batteries using **MV** as the negolyte. These 100% SOC electrolytes were then isolated from their respective cells and stored under the same conditions as the previously discussed batteries, that is, an inert atmosphere, the same temperature, the same humidity level, and a strictly identical storage time.

Analyses of the 100% SOC electrolytes after 7 days of storage showed a current intensity decrease of 19% and 13% for **PPO** and **4‐TMA‐TEMPO**, respectively, as measured by CV (Figure S11). These experiments report a loss of electrochemically active species concentrations, clearly indicating that the degradation of nitroxides is independent of battery cycling. It is confirmed by ^1^H NMR analyses of aged posolyte showing peaks at 1.58, 1.83, 2.37, 7.99, and 8.81 ppm for **PPO**, with signal intensities nearly the same as for the cycled electrolyte (Figure 7).

## 3 Conclusion

An efficient and general method for the catalytic preparation of substituted isoindoline and 2,3‐dihydro‐1H‐pyrrolo[3,4‐c]pyridine‐based nitroxides is described with high functional compatibility and using diverse unactivated triple bonds and α,ω‐diynes. The scope of the reaction is broader than methods usually reported to access this family of nitroxides.

The electrochemical properties of two nitroxide posolytes, a cationic (**PPO**) and an anionic (**TC‐TMIO**), were evaluated, revealing an enhanced oxidation potential (+220 mV) for **PPO** compared to the benchmark **4‐TMA‐TEMPO**.

AORFB has been constructed using **PPO** as a posolyte and **MV** as a negolyte at neutral pH and displays a significantly higher cell voltage of 1.56 V regarding other reported nitroxide posolytes, and represents a 16% improvement regarding the **MV**/**4‐TMA‐TEMPO** system. The higher solubility of **PPO** offers further improvements in energy density, potentially reaching 57 Wh L^−1^ due to a theoretical capacity of 80 Ah L^−1^. Cycling results for the **MV**/**PPO** battery showed a capacity loss of 3.22%, compared to 1.01% for the **MV**/**4‐TMA‐TEMPO** system. Postmortem studies of cycled electrolytes and electrolytes stored outside the battery at 100% SOC revealed that the degradation of **PPO** is primarily due to the oxidized form of the molecules. Despite the less‐than‐optimal stability of **PPO**, our results constitute a crucial step in the development of new redox‐active molecules that are easily tailored for high energy and power densities. The proposed synthetic method and the interesting structural and redox properties of **PPO** open new perspectives and will enable further progress in the energy storage field.

## Supporting Information

Additional supporting information can be found online in the Supporting Information section. **Supporting** **Scheme S1** **:** Synthesis of bis(2‐méthylbut‐3‐yn‐2‐yl) amine α, ω‐diynes (**1**). **Supporting** **Scheme S2** **:** Synthesis of 1,1,3,3,6‐pentamethyl‐2,3‐dihydro‐1H‐pyrrolo[3,4‐c] pyridine (**5**). **Supporting** **Scheme S3** **:** Synthesis of 1,1,3,3,6‐péntamethyl‐1,3‐dihydro‐2H‐pyrrolo[3,4‐c] pyridin‐2‐oxyl (**6**). **Supporting** **Scheme S4** **:** Synthesis of 1,1,3,3,6,7‐hexaaméthyl‐1,3‐dihydro‐2H‐pyrrolo[3,4‐c] pyridinium‐2‐oxyl chloride (**PPO**). **Supporting** **Scheme S5:** Synthesis of dimethyl 4,4′‐azanediylbis(4‐methylpent‐2‐ynoate). **Supporting** **Scheme S6:** Synthesis of 5,6‐diethyl 4,7‐dimethyl 1,1,3,3‐tetramethylisoindoline‐4,5,6,7‐tetracarboxylate ( **5b** ). **Supporting** **Scheme S7** **:** Synthesis of 5,6‐diethyl 4,7‐dimethyl 2‐oxyl‐1,1,3,3‐tetramethylisoindoline‐4,5,6,7‐tetracarboxylate ( **7** ). **Supporting** **Scheme S8** **:** Synthesis of 1,1,3,3‐tetramethylisoindoline2‐oxyl‐4,5,6,7‐tetracarboxylate de sodium (**TC‐TMIO**). **Supporting** **Fig.** **S1:** ^1^H NMR spectrum (400 MHz, DMSO) of bis(2‐methylbut‐3‐yn‐2‐yl) amine (**1**). **Supporting** **Fig.** **S2:** ^13^C APT NMR spectrum (75 MHz, DMSO) of bis(2‐methylbut‐3‐yn‐2‐yl) amine (**1**). **Supporting** **Fig.** **S3:** ^1^H NMR spectrum (300 MHz, CDCl3) of 1,1,3,3,6‐pentamethyl‐2,3‐dihydro‐1H‐pyrrolo[3,4‐c]pyridine (**5**). **Supporting** **Fig.** **S4:** ^13^C APT NMR spectrum (75 MHz, CDCl3) of 1,1,3,3,6‐pentamethyl‐2,3‐dihydro‐1H‐pyrrolo[3,4‐c]pyridine (**5**). **Supporting** **Fig.** **S5:** N ^1^H NMR spectrum (300 MHz, MeOD, + ascorbic acid) of 1,1,3,3,6‐pentamethyl‐1,3‐dihydro‐2H‐pyrrolo[3,4‐c]pyridin‐2‐oxyl ( **6** ). **Supporting** **Fig.** **S6:** ^13^C NMR spectrum ‐ APT (75 MHz, MeOD, + ascorbic acid) of 1,1,3,3,6‐pentamehyl‐1,3‐dihydro‐2H‐pyrrolo[3,4‐c]pyridin‐2‐oxyl. **Supporting** **Fig.** **S7:** ^1^H NMR spectrum (300 MHz, D2O+ ascorbic acid) 1,1,3,3,6,7‐hexaamethyl‐1,3‐dihydro‐2H‐pyrrolo[3,4‐c]pyridinium‐2‐oxyl chloride (1) (**PPO**). **Supporting** **Fig.** **S8:** ^13^C ‐ APT NMR spectrum (75 MHz, D2O + ascorbic acid) of 1,1,3,3,6,7‐hexaamethyl‐1,3‐dihydro‐2H‐pyrrolo[3,4‐c]pyridinium‐2‐oxyl chloride (1) (**PPO**). **Supporting** **Fig.** **S9:** High resolution mass spectrum (ESI^+^) of N,N,N,2,2,5,5‐heptamethylpyrrolidinyloxy‐3‐ammonium chloride (2) (**PPO**). Expected ion at m/z 200,1886. **Supporting** **Fig.** **S10:** ^1^H NMR spectrum (400 MHz, CDCl_3_) of dimethyle 4,4′‐azanediylbis(4‐methylpent‐2‐ynoate) ( **2** ). **Supporting** **Fig.** **S11:** ^13^C ‐ APT NMR spectrum (101 MHz, CDCl_3_) of dimethyle 4,4′‐azanediylbis(4‐methylpent‐2‐ynoate) ( **2** ). **Supporting** **Fig.** **S12:** ^1^H NMR Spectrum (400 MHz, CDCl_3_) of 5,6‐diethyl 4,7‐dimethyl 1,1,3,3‐tetraméthylisoindoline‐4,5,6,7‐tetracarboxylate ( **3** ). **Supporting** **Fig.** **S13:** ^13^C – APT NMR spectrum (101 MHz, CDCl_3_) of 5,6‐diethyl 4,7‐dimethyl 1,1,3,3‐tetramethylisoindoline‐4,5,6,7‐tetracarboxylate ( **3** ). **Supporting** **Fig.** **S14:** ^1^H NMR spectrum (300 MHz, D2O) of 1,1,3,3‐tetramethylisoindoline2‐oxyl‐4,5,6,7‐tetracarboxylate de sodium (**TC‐TMIO**). **Supporting** **Fig.** **S15:** ^13^C ‐ APT NMR spectrum (75 MHz, D_2_O) of 1,1,3,3‐tetramethylisoindoline2‐oxyl‐4,5,6,7‐tetracarboxylate de sodium (**TC‐TMIO**). **Supporting** **Fig.** **S16:** High resolution mass spectrum (ESI^+^) of 1,1,3,3‐tetramethylisoindoline2‐oxyl‐4,5,6,7‐tetracarboxylate de sodium (**TC‐TMIO**). Expected ion at m/z 476,9994. **Supporting** **Fig.** **S17:** (a) Cyclic voltammograms of PPO (1) recorded at various scan rates (10–700 mV·s^−1^). (b) Plot of the anodic peak current (Ip,ox) versus the square root of the scan rate (ν^1/2^). (c) Plot of ψ as a function of ν^−1/2^. Conditions: 5 mM analyte in 1 M NaCl supporting electrolyte; working electrode: glassy carbon (area = 0.0706 cm^2^); counter electrode: platinum wire; reference electrode: Ag/AgCl (saturated KCl). **Supporting** **Fig.** **S18:** (a) Cyclic voltammograms of TC‐TMIO recorded at various scan rates (10–700 mV·s^−1^). (b) Plot of the anodic peak current (Ip,ox) obtained from CV as a function of the square root of the scan rate (ν^1/2^). (c) Plot of ψ versus ν^−1/2^. Conditions: 5 mM analyte in 1 M NaCl supporting electrolyte; working electrode (WE): glassy carbon (area = 0.0706 cm^2^); counter electrode (CE): platinum; reference electrode (REF): Ag/AgCl (saturated KCl). **Supporting** **Fig.** **S19:** a) Cyclic voltammograms of **4‐TMA TEMPO** at various scan rates (10 to 700 mV s^−1^). b) Plot of Ip over the square root of scan rates for **4‐TMA TEMPO** at various scan rates (10 to 700 mV s^−1^). c) Plot of over for **4‐TMA TEMPO** at various scan rates (10 to 700 mV s^−1^). Conditions: 5 mM analyte in 1 M NaCl (aq) electrolyte; glassy carbon working electrode; platinum counter electrode; Ag/AgCl reference electrode. **Supporting** **Fig.** **S20:** (a) Rotating disk electrode (RDE) voltammograms of PPO (1) recorded at various rotation rates (400–2000 rpm). (b) Plot of I_la_ (measured at 0.99 V) versus the square root of the rotation rate (ω^1/2^). (c) Koutecky–Levich plots of I_K−1_ versus ω^−1/2^. (d) Tafel plot of overpotential (*η*) versus log I_K(*η*)_. Conditions: 5 mM analyte in 1 M NaCl supporting electrolyte; rotating glassy carbon working electrode (area = 0.1963 cm^2^); platinum counter electrode; Ag/AgCl (saturated KCl) reference electrode. **Supporting** **Fig.** **S21:** a) Linear sweep voltammograms of **4‐TMA TEMPO** at various rotating rates (400 to 2000 rpm); b) Levich plots of the limiting current (measured at 1.25 V) versus the square root of rotation rates for **4‐TMA TEMPO**; c) i^−1^ versus ω^−1/2^ for **4‐TMA TEMPO**; d) Overpotential versus the logarithm of kinetic current and the corresponding fitted Tafel plots for **4‐TMA TEMPO**. Conditions: 5 mM analyte in 1 M NaCl (aq) electrolyte; working electrode glassy carbon rotating disk electrode; platinum counter electrode; Ag/AgCl reference electrode. **Supporting** **Fig.** **S22:** Charge/discharge capacity vs cycle number profiles for MV/**4TMA TEMPO** batteries presented in figure 2. Duration: 6.8 days. **Supporting** **Fig.** **S23:** Charge/discharge capacity curves as a function of voltage of **PPO/MV** flow battery. Conditions: anolyte: 75 ml of 0.1 M **MV** in 1 M NaCl (aq); catholyte: 25 ml of 0.1 M PPO in 1M NaCl (aq); Fumasep FAA‐3‐50 anion‐exchange membrane; current density 10 mA cm^−2^; temperature 23°C, time 6.8 days. **Supporting** **Fig.** **S24:** Polarization curves at 50% SOC of the MV/PPO redox flow battery: (a) charge; (b) discharge. **Supporting** **Fig.** **S25:** Cyclic voltammograms of the MV (a) and PPO (b) electrolytes before and after cycling. **Supporting** **Fig.** **S26:** (a) UV–vis spectra of PPO (1) at various concentrations. (b) Calibration curve of PPO (1). **Supporting** **Fig.** **S27:** a) ^1^H NMR spectra of the 4‐TMA TEMPO electrolyte before and after cycling, and after being stored for 6.8 days in the oxidized state (100% SOC). b) UV‐visible absorbance spectra of posolytes containing 4‐TMA TEMPO before(plain) and after (dashed) cycling. **Supporting** **Table** **S1:** Experimentally derived average capacity fade rates using linear regression for both 4‐TMA TEMPO/MV flow battery experiment and the PPO/MV flow battery experiment.

## Conflicts of Interest

The authors declare no conflicts of interest.

## Supporting information

Supplementary Material

## Data Availability

CCDC number 2513657 (for **TC‐TMIO**), contains the supplementary crystallographic data for this paper. These data are provided free of charge by the joint Cambridge Crystallographic Data Centre and Fachinformationszentrum Karlsruhe Access Structures service.
