# Supplementary material for: High Potential Isoindoline‐Based Nitroxides Posolytes for Aqueous Organic Redox Flow Batteries
Source: ChemSusChem. 2026 Feb 1;19(3):e202502461. doi: 10.1002/cssc.202502461 (PMC12861492; doi:10.1002/cssc.202502461)
Supplement: Supplementary file 1 — Supplementary Material [file CSSC-19-e202502461-s001.pdf]

HIGH POTENTIAL ISO-INDOLINE-BASED NITROXIDES AS POSOLYTES FOR AQUEOUS ORGANIC REDOX FLOW BATTERIES

Karim Boutamine,[a,b,c], Gilles Casano,[a] Patricia Bassil[b,c], Sebastien Gauden,[a] Cecilia Poderi,[a] Emilie Pepe,[a] Frédéric Favier[b,c], Steven Le Vot*[b,c] and Olivier Ouari*[a,c]

Supplementary info

All chemicals used were purchased from Aldrich Chemical Co. and TCI. Purification of products was accomplished by flash chromatography on silica gel (Merck silica gel, 60, 230-400 mesh). NMR spectra were recorded on a Bruker AVL 300 spectrometer (1H NMR 300.1 MHz and 13C NMR 75.5 MHz) using $CDCl_3$ as the solvent (internal reference). Mass spectral analyses were carried out using a Q-STAR Elite at the Aix-Marseille University Mass Spectrum Facility, Spectropole Saint-Jérôme. EPR measurements were performed on a Bruker Elexsys spectrometer operating at 9.4 GHz (X-band) in 50 µL capillaries using the following parameters, microwave power 5mW and modulation amplitude 0.4 G. The final products were purified to ≥ 95% and were confirmed by HPLC-MS analysis. HPLC-MS experiments were performed using an Agilent 1260 infinity system coupled with a 6120 simple quadrupole. This system was equipped with a C18 column (Zorbax 1.8 µM, 3 x 50 mm) that was equilibrated with 10% vol. acetonitrile in 0.1% vol. formic acid aqueous solution at the flow rate of 0.21 mL/min.

Synthesis of PPO

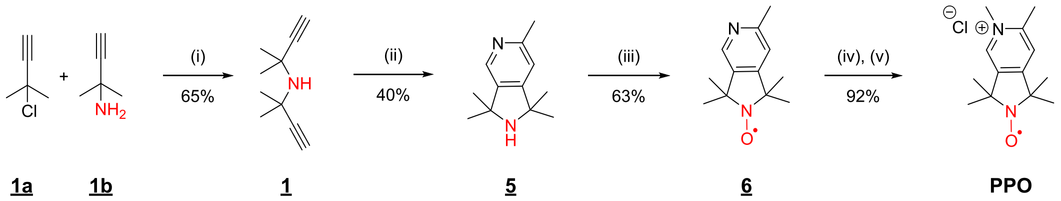

(i) Cu powder (1%), $CuCl_2$ (3%), DMF, 48h, 25°C ; (ii) $CoCl_2$. 6 H_2O (10%), dppe (12%), Zn powder (20%), MeCN, 48h, 60°C ; (iii) $MeCO_3H$ (0,9 eq), K_2CO_3 (0,9 eq), H_2O/DCM (10 :7), 3h, 25°C ; (iv) MeI (5 eq), MeCN, 24h à 50°C ; (v) Amberlite IRA-900 chloride form).

Synthesis of PPO (1) :

Synthesis of bis(2-methylbut-3-yn-2-yl) amine (1a) :

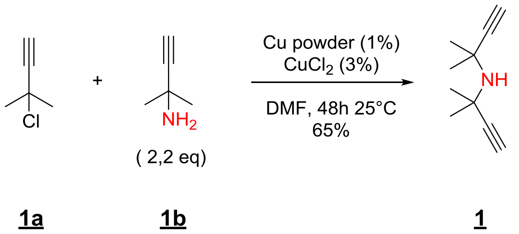

***Scheme 1** : Synthesis of bis(2-méthylbut-3-yn-2-yl) amine α, ω-diynes (1).*

In a 250 mL round-bottom flask equipped with a magnetic stir bar, 0.13 g (0.97 mmol) of $CuCl_2$ and 0.18 g (0.29 mmol) of copper (0) were dissolved in 50 mL of anhydrous DMF. Subsequently, 17.83 g

(214.52 mmol) of 2-methylbut-3-yn-2-amine (**1a**) and 10.00 g (97.50 mmol) of 3-chloro-3-methylbut-1-yne (**1b**) were added. The reaction mixture was stirred at 25 °C for 48 hours, and the reaction progress was monitored by TLC. After 48 hours, 100 mL of water and 25 mL of a 20% aqueous NaOH solution were added. The resulting suspension was filtered, and the filtrate was transferred into a separatory funnel. The aqueous phase was extracted five times with 200 mL of diethyl ether. The combined organic extracts were dried over Na_2SO_4. After filtration, the solvent was removed under reduced pressure using a rotary evaporator. Purification by distillation at 132 °C under 10–20 mbar afforded 9.50 g of a clear oil, corresponding to a yield of 65%.

^{1}H NMR (400 MHz, DMSO) : **δ** (ppm) 2.99 (s, 2H), 2.10 (s, 1H), 1.43 (s, 12H).

^{13}C NMR - APT (75 MHz, DMSO): **δ** (ppm) 91.76 (C), 72.36 (CH), 48.48 (C), 32.53 (CH_3).

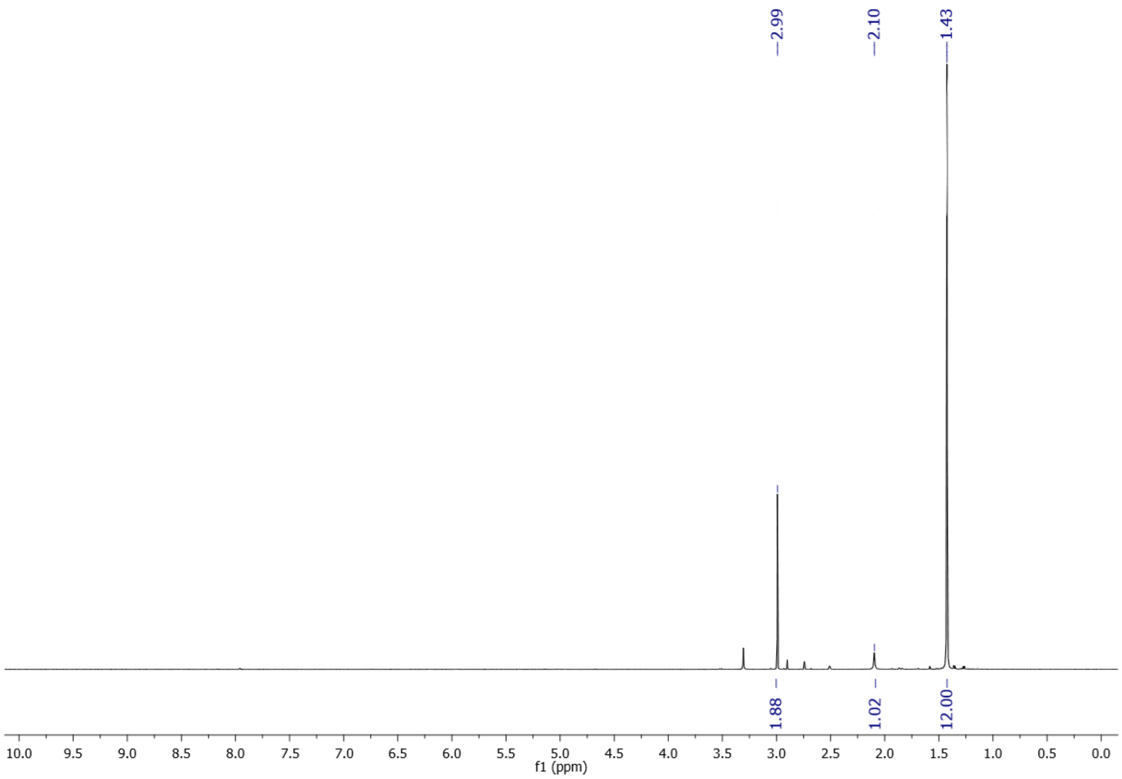

Figure S1: *^{1}H NMR spectrum (400 MHz, DMSO) of bis(2-methylbut-3-yn-2-yl) amine (**1**).*

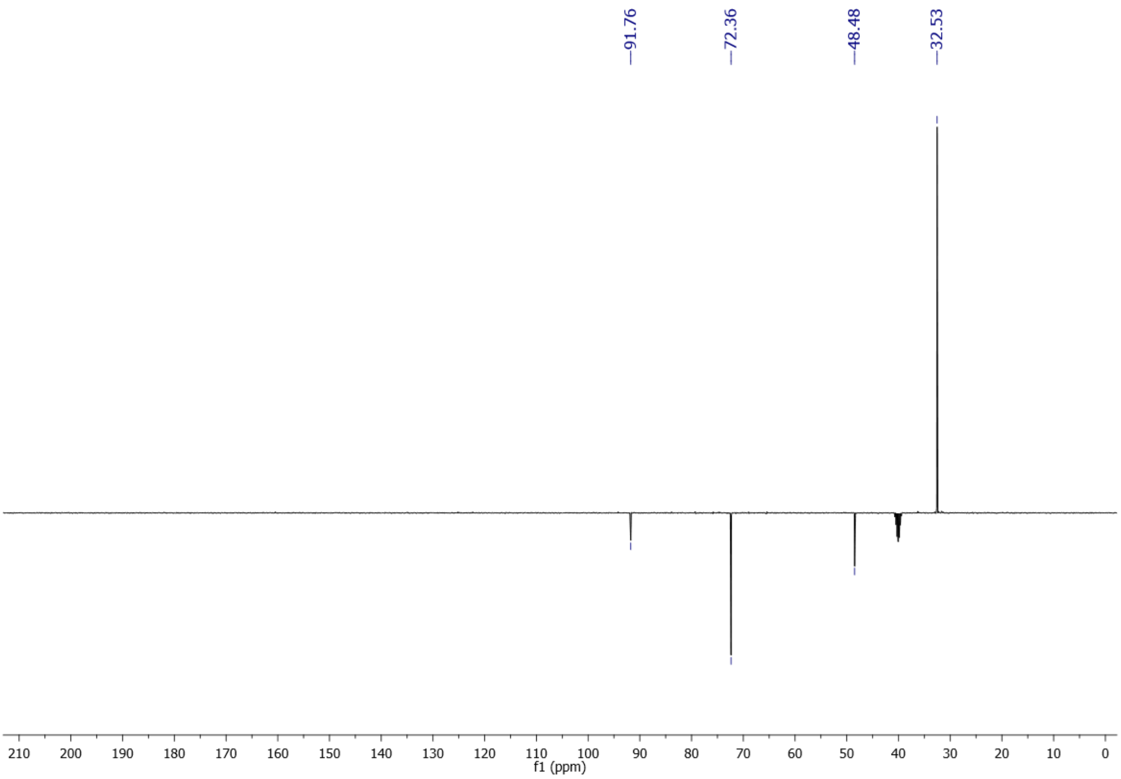

Figure S2: *^{13}C APT NMR spectrum (75 MHz, DMSO) of bis(2-methylbut-3-yn-2-yl) amine (**1**).*

Synthesis of 1,1,3,3,6-pentamethyl-2,3-dihydro-1H-pyrrolo[3,4-c] pyridine :

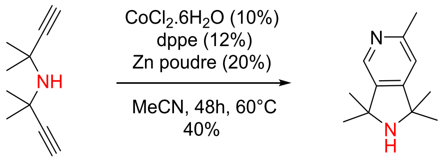

1 **5**

***Scheme 21** : Synthesis of 1,1,3,3,6-pentamethyl-2,3-dihydro-1H-pyrrolo[3,4-c] pyridine (**5**).*

In a 250 mL three-necked flask equipped with a magnetic stirrer and a condenser, 5.00 g (33.50 mmol) of bis(2-methylbut-3-yn-2-yl)amine α,ω-diynes (**1**) and 0.43 g (6.70 mmol) of zinc were dissolved in 80 mL of anhydrous acetonitrile. Using a syringe, a degassed solution containing 1.60 g (4.02 mmol) of dppe and 0.79 g (3.35 mmol) of $CoCl_2 \cdot 6H_2O$ in 80 mL of anhydrous acetonitrile was added. The reaction mixture was then heated at 60 °C for 48 hours. After this time, the suspension was cooled in an ice bath and filtered. The solvent was removed under reduced pressure, and the crude residue was purified by chromatography (eluent: AcOEt/Pentane/Acetone), affording 2.61 g of colorless crystals in 40% yield.

^{1}H NMR (300 MHz, $CDCl_3$) : **δ** (ppm) 8.23 (m, 1H), 6.86 (s, 1H), 2.51 (s, 3H), 2.02 (s, 1H), 1.42 (s, 6H), 1.38 (s, 6H).

^{13}C NMR (75 MHz, $CDCl_3$) : δ (ppm) 158.47 (Cq), 156.82 (Cq), 142.87 (CH), 141.61 (Cq), 115.98 (CH), 62.65 (Cq), 61.96 (Cq), 31.85 (CH_3), 31.16 (CH_3), 24.25 (CH_3).

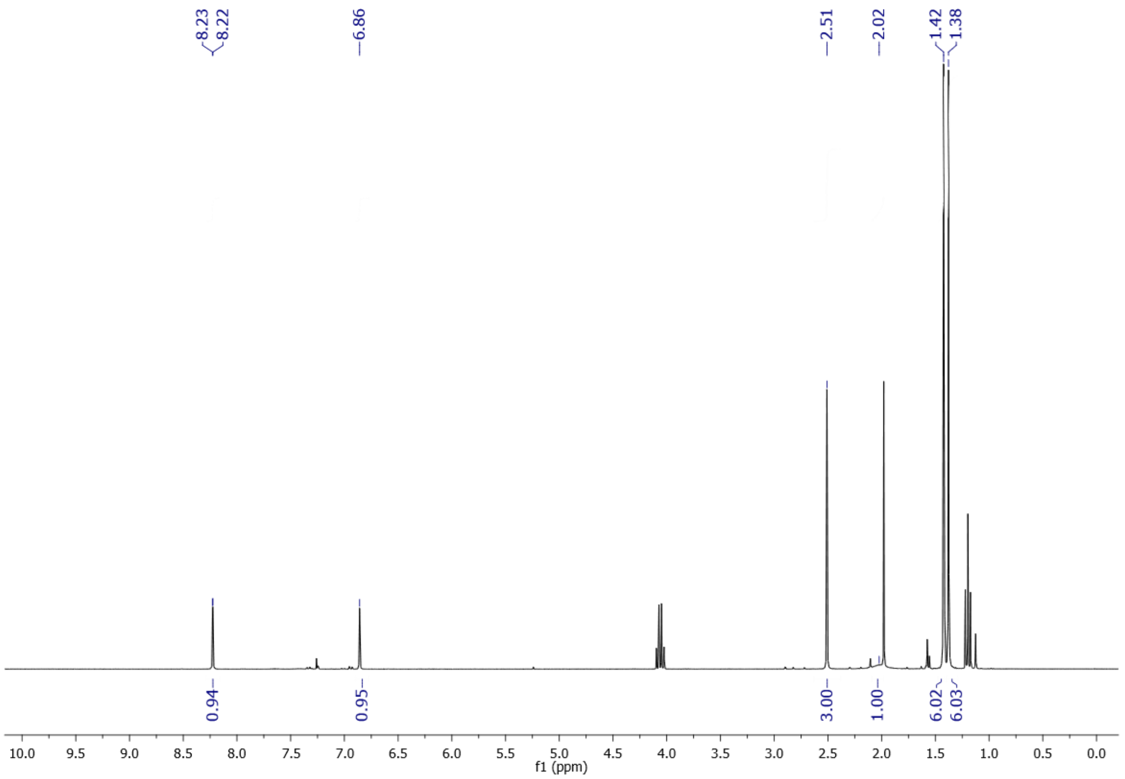

Figure S3: *^{1}H NMR spectrum (300 MHz, $CDCl_3$) of 1,1,3,3,6-pentamethyl-2,3-dihydro-1H-pyrrolo[3,4-c]pyridine (5).*

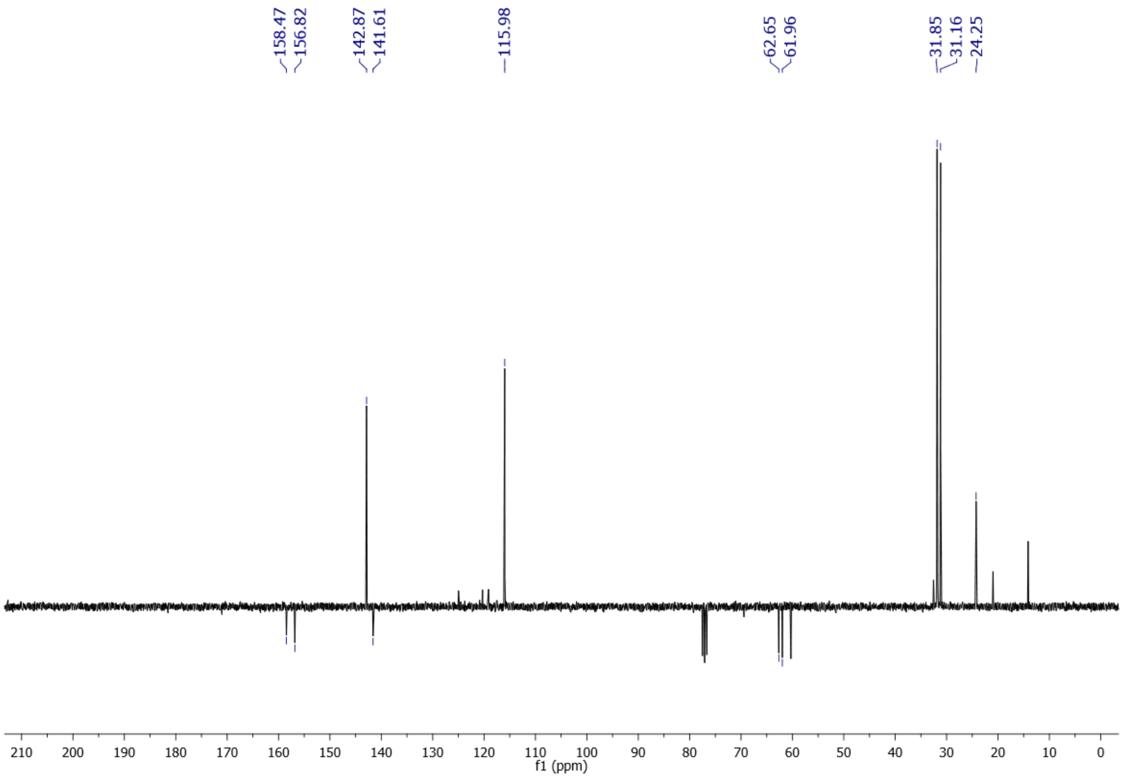

Figure S4: *^{13}C APT NMR spectrum (75 MHz, $CDCl_3$) of 1,1,3,3,6-pentamethyl-2,3-dihydro-1H-pyrrolo[3,4-c]pyridine (5).*

Synthesis of 1,1,3,3,6-pentamethyl-1,3-dihydro-2H-pyrrolo[3,4-c] pyridin-2-oxyl (6) :

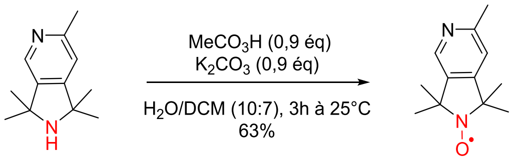

5 6

***Scheme 3** : Synthesis of 1,1,3,3,6-péntamethyl-1,3-dihydro-2H-pyrrolo[3,4-c] pyridin-2-oxyl (6).*

In a 500 mL three-necked flask equipped with a magnetic stirrer and placed in an ice bath, 2.60 g (13.66 mmol) of 1,1,3,3,6-pentamethyl-2,3-dihydro-1H-pyrrolo\[3,4-c]pyridine (5) were dissolved in a mixture of 70 mL dichloromethane and 100 mL water. Under vigorous stirring, 2.67 g (12.30 mmol) of peracetic acid (35% $MeCO_3H$ in acetic acid) and 4.86 g (12.30 mmol) of a 35% K_2CO_3 solution were slowly added. After the addition, the reaction mixture was stirred at 25 °C for 2 hours. The mixture was then transferred to a separatory funnel. The organic phase was separated, and the aqueous phase was extracted three times with 100 mL dichloromethane. The combined organic layers were dried over Na_2SO_4. After filtration, the solvent was evaporated under reduced pressure, affording 1.77 g of a yellow solid after chromatographic purification (system: SiO_2, Pentane/AcOEt), with a yield of 63%.

^{1}H NMR (300 MHz, MeOD, addition of ascorbic acid) : **δ** (ppm) 8.19 (s, 1H), 7.12 (s, 1H), 2.84 (s, 1H), 2.51 (s, 3H), 1.39 (s, 6H), 1.37 (s, 6H).

^{13}C NMR (75 MHz, MeOD, addition of ascorbic acid) : **δ** (ppm) 156.64 (Cq), 156.54 (Cq), 141.76(CH), 139.36(Cq), 117.06 (CH), 66.39 (Cq), 65.27 (Cq), 25.53 (CH_3), 24.97 (CH_3), 22.69 (CH_3).

EPR (9 GHz, 2 mM in MeOH, 20°C) **:** a_N = 1,46 mT, 3 lines.

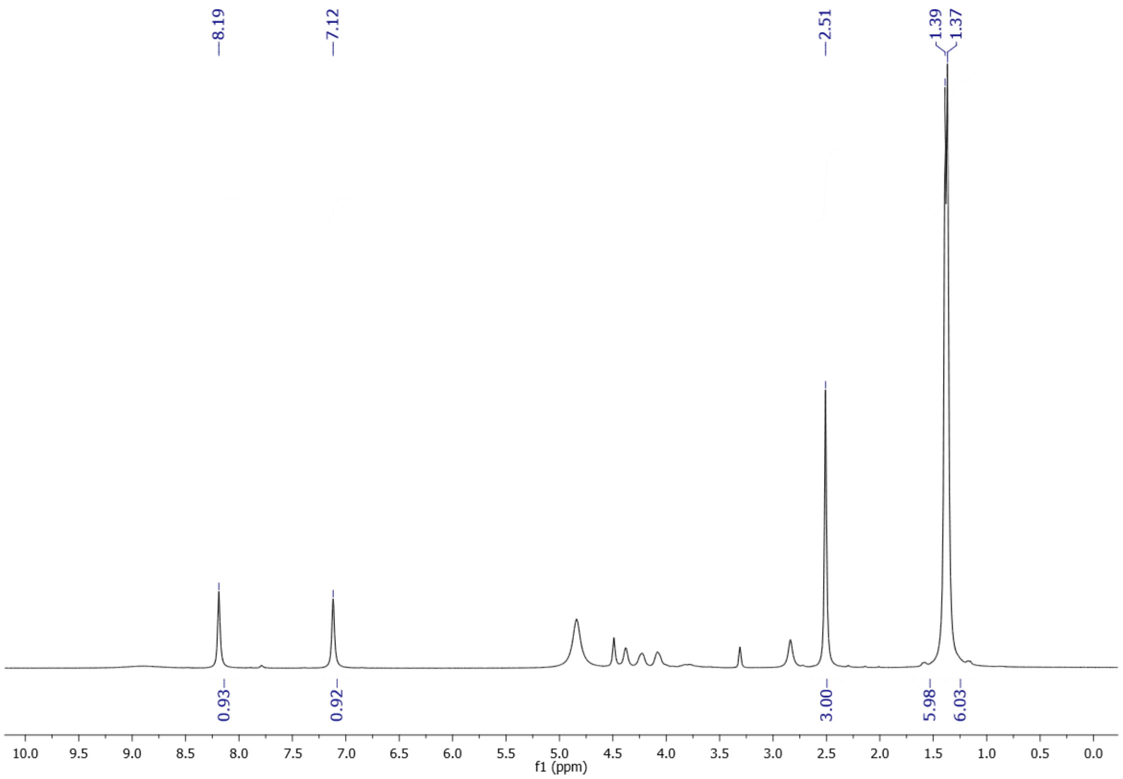

Figure S5: *N* [1]*H NMR spectrum (300 MHz, MeOD, + ascorbic acid) of 1,1,3,3,6-pentamethyl-1,3-dihydro-2H-pyrrolo[3,4-c]pyridin-2-oxyl (**6**).*

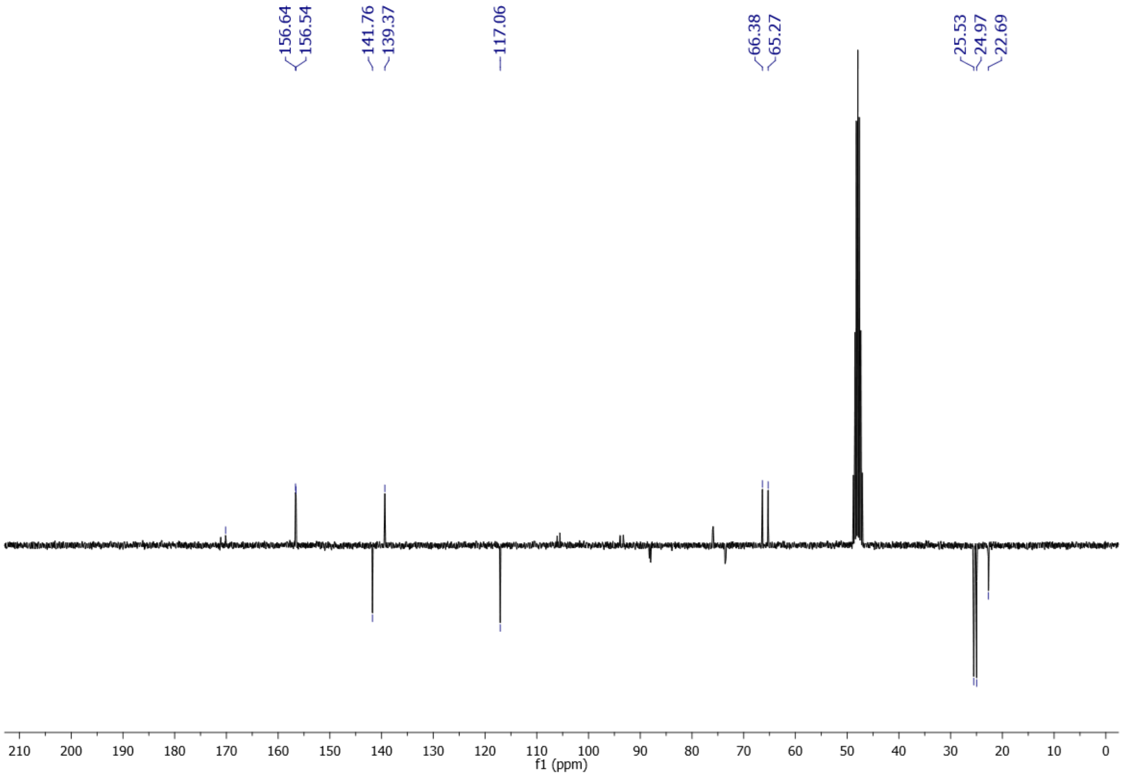

Figure S6: *^{13}C NMR spectrum - APT (75 MHz, MeOD, + ascorbic acid) of 1,1,3,3,6-pentamehyl-1,3-dihydro-2H-pyrrolo[3,4-c]pyridin-2-oxyl*

Synthesis of 1,1,3,3,6,7-hexaamethyl-1,3-dihydro-2H-pyrrolo[3,4-c] pyridinium-2-oxyl chloride (PPO) :

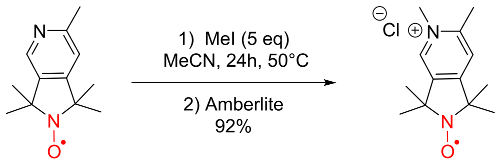

6 **PPO**

Scheme 4 *: Synthesis of 1,1,3,3,6,7-hexaaméthyl-1,3-dihydro-2H-pyrrolo[3,4-c] pyridinium-2-oxyl chloride (**PPO**).*

In a 250 m L three-necked flask equipped with a magnetic stirrer and a condenser, 1.77 g (8.62 mmol) of 1,1,3,3,6-pentamethyl-1,3-dihydro-2H-pyrrolo\[3,4-c]pyridin-2-oxyl (**6**) were dissolved in 50 mL of MeCN. Then, 2.66 mL (43.11 mmol) of iodomethane were added dropwise, and the reaction mixture was stirred at 50 °C for 24 h. After 24 hours, the solvent was removed under reduced pressure, and 30 mL of water were added. The aqueous phase was extracted twice with 50 mL of ethyl acetate (AcOEt) using a separatory funnel. The aqueous solution was subsequently passed through a chloride-form Amberlite IRA-900 chloride form anion-exchange resin. Freeze drying of the solution afforded 2.03 g of a yellow solid in 92% yield.

^{1}H NMR (300 MHz, MeOD, addition of ascorbic acid) : **δ** (ppm) 8.59 (s, 1H), 7.75 (s, 1H), 4.10 (s, 3H), 2.67 (s, 3H), 1.43 (s, 12H).

^{13}C NMR (75 MHz, MeOD, addition of ascorbic acid) : **δ** (ppm) 162.09 (Cq), 156.01 (Cq), 141.72 (Cq), 140.30 (CH), 122.98 (CH), 69.22 (Cq), 67.79 (Cq), 45.52 (CH_3), 24.96 (CH_3), 24.32 (CH_3), 19.94 (CH_3).

ESI-MS : predicted [M] 255,1264 Da, $[M\text{-}Cl^-]^+$ 220.1570 Da. Predicted agglomerate ion $[2M - Cl^-]^+$ 475.2834.

Detected exact mass [M] 255,1264 Da, $[M\text{-}Cl^-]^+$ 220.2000 Da. Agglomerate ion $[2M - Cl^-]^+$ 475.2834 Da.

EPR (9 GHz, 1 mM in water, 20°C) **:** a_N = 1,53 mT, 3 lines.

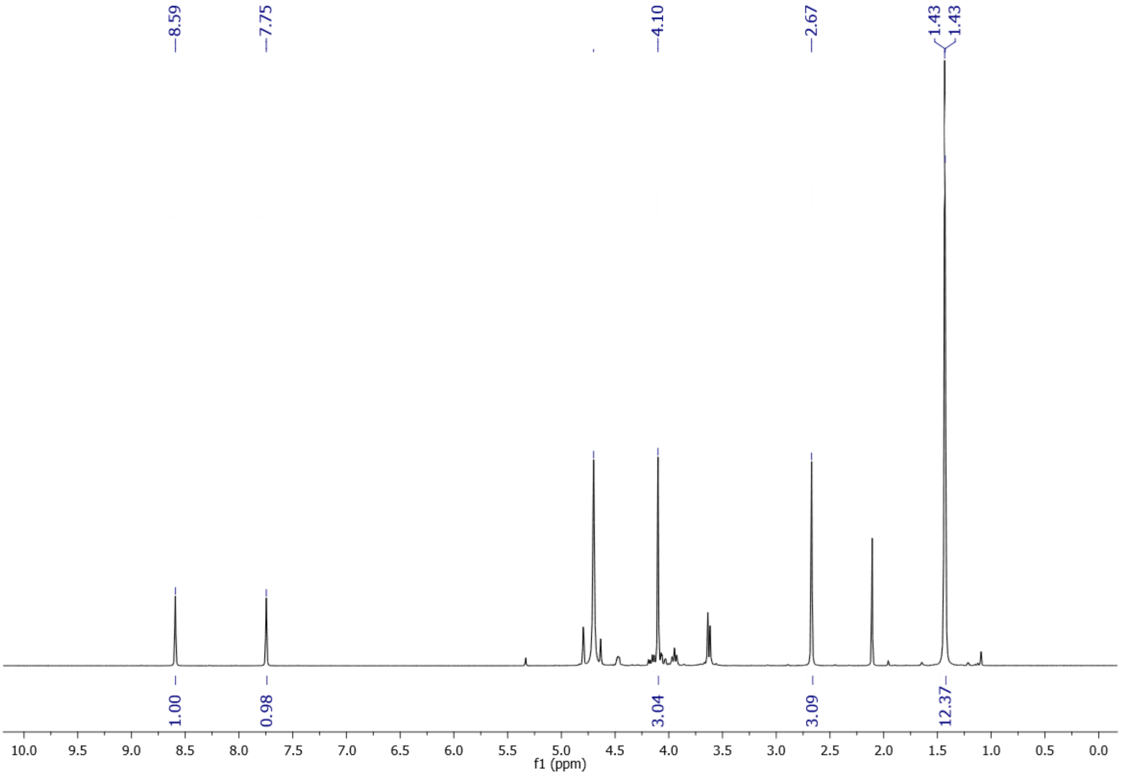

Figure S7: *^{1}H NMR spectrum (300 MHz, D_2O+ ascorbic acid) 1,1,3,3,6,7-hexaamethyl-1,3-dihydro-2H-pyrrolo[3,4-c]pyridinium-2-oxyl chloride (1) (**PPO**).*

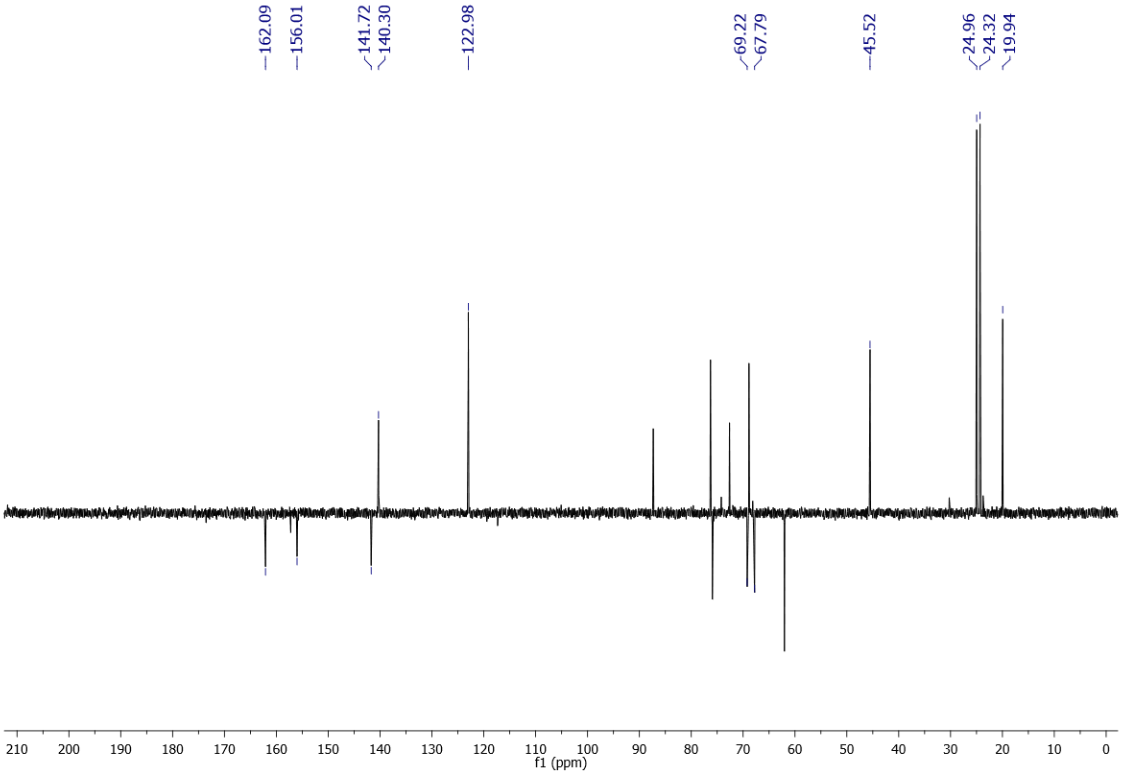

Figure S8: *^{13}C - APT NMR spectrum (75 MHz, D_2O + ascorbic acid) of 1,1,3,3,6,7-hexaamethyl-1,3-dihydro-2H-pyrrolo[3,4-c]pyridinium-2-oxyl chloride (1) (**PPO**).*

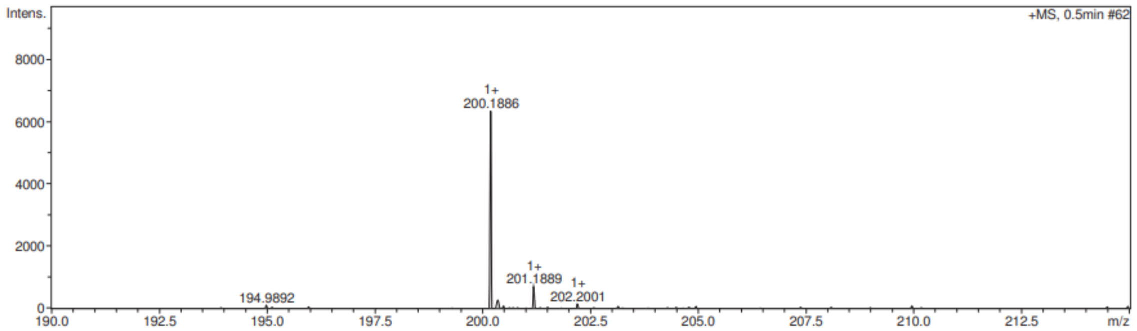

Meas. m/z	#	Ion Formula	Score	m/z	err [mDa]	err [ppm]	mSigma	rdb	e⁻ Conf	N-Rule	Adduct
200.1886	1	C11H24N2O	100.00	200.1883	0.3	1.3	13.7	1.0	odd	ok	M+H

Figure S9: *High resolution mass spectrum (ESI$^+$) of N,N,N,2,2,5,5-heptamethylpyrrolidinyloxy-3-ammonium chloride (2) (PPO). Expected ion at m/z 200,1886.*

Synthesis of TC-TMIO :

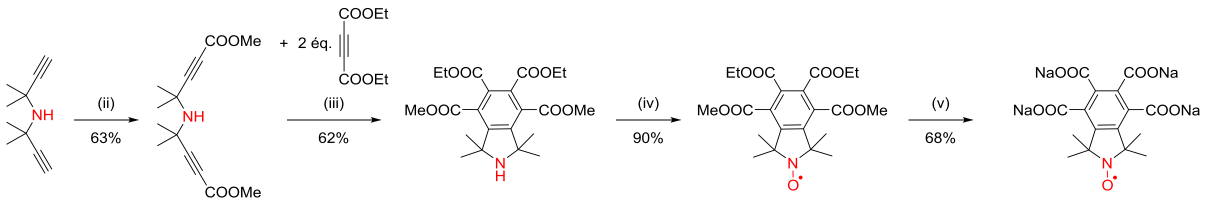

1 2 3 7 **TC-TMIO**

(i) Cu powder (1%), $CuCl_2$ (3%), DMF, 48h, 25°C (ii) nBuLi (2,5 eq), ClCOOMe (2 eq.), THF, -78°C then 25°C for 24h ; (iii) $(PPh_3)_3RhCl$ (5%), toluene, 24h, 85°C ; (iv) $MeCO_3H$ (3 eq), K_2CO_3 (3 eq), H_2O/DCM (1 :1), 3h, 25°C ; (v) NaOH (excess), EtOH/ H_2O (3 :2), 24h, 80°C.

Synthesis of dimethyle 4,4'-azanediylbis(4-methylpent-2-ynoate) :

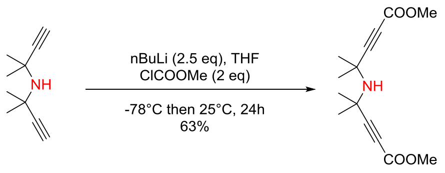

1 2

Scheme 5*: Synthesis of dimethyl 4,4'-azanediylbis(4-methylpent-2-ynoate)*

In a 250 mL flask equipped with a magnetic stirrer, 2.00 g (13.40 mmol) of bis(2-methylbut-3-yn-2-yl)amine (**1**) were dissolved in 170 mL of anhydrous THF. The reaction mixture was cooled to –78 °C using an acetone/liquid nitrogen bath. Then, 13.40 mL (33.50 mmol) of a 2.5 M nBuLi solution in hexane were added dropwise via syringe. Thirty minutes after completion of the addition, the reaction temperature was raised to 25 °C, and 2.10 mL (26.80 mmol) of methyl chloroformate were added dropwise. The mixture was stirred at 25 °C for 24 hours. The suspension was cooled in an ice bath, followed by the addition of 150 mL of saturated aqueous NH_4Cl solution. The reaction mixture was

transferred to a separatory funnel and extracted twice with 200 mL AcOEt. The combined organic phases were dried over Na_2SO_4. After filtration, the solvent was removed under reduced pressure, affording 2.26 g of a viscous oil after chromatographic purification (system: SiO_2, Pentane/AcOEt 9:1), corresponding to a yield of 63%.

^{1}H NMR (300 MHz, $CDCl_3$) : **δ** (ppm): 3.68 (s, 6H), 1.52 – 1.42 (m, 13H).
^{13}C NMR (75 MHz, $CDCl_3$) : **δ** (ppm) 154.17 (C), 93,28 (C), 74.84 (C_q), 52.46 (CH_3), 48.90 (C), 31.28 (CH_3).

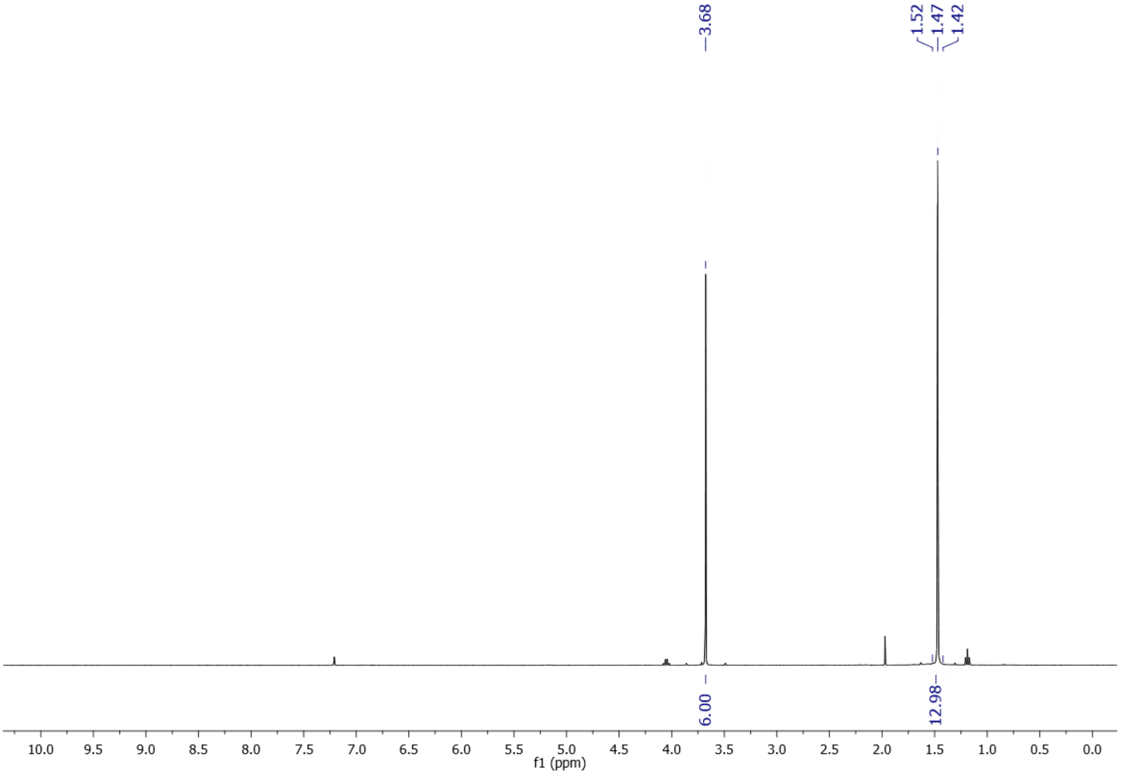

Figure S10: *^{1}H NMR spectrum (400 MHz, $CDCl_3$) of dimethyle 4,4'-azanediylbis(4-methylpent-2-ynoate) (__2__).*

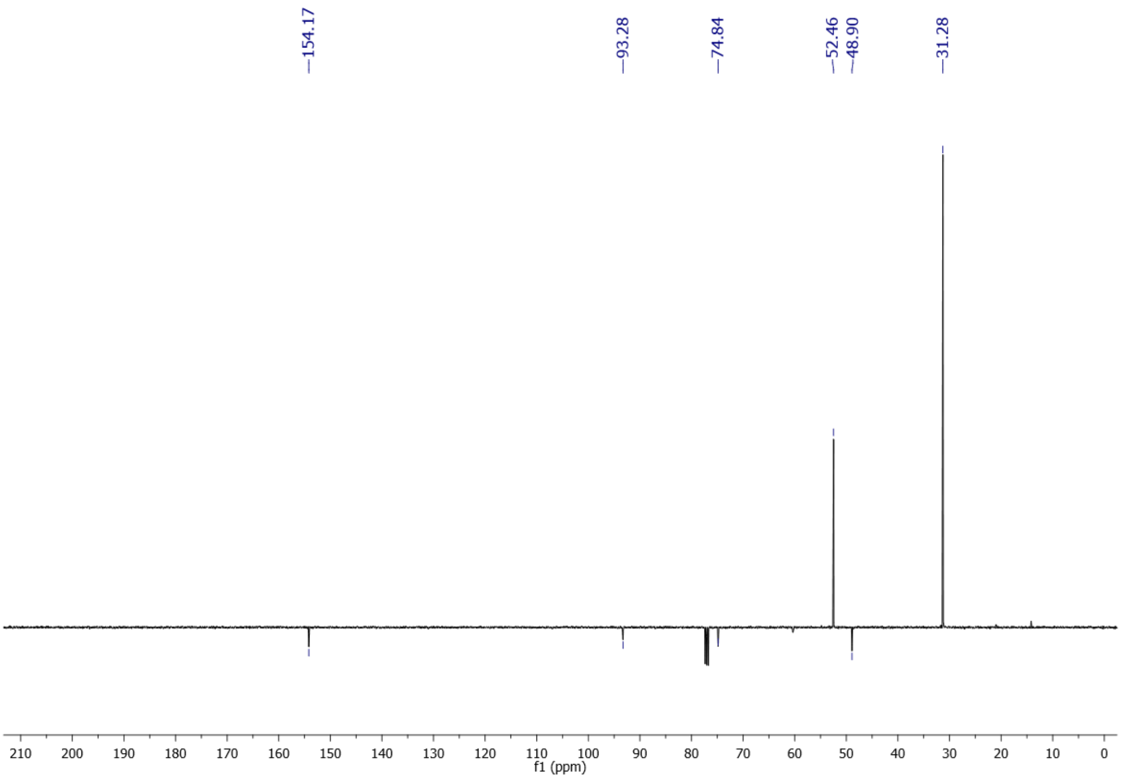

Figure S11: *^{13}C - APT NMR spectrum (101 MHz, $CDCl_3$) of dimethyle 4,4'-azanediylbis(4-methylpent-2-ynoate) (2)*

Synthesis of 5,6-diethyl 4,7-dimethyl 1,1,3,3-tetramethylisoindoline-4,5,6,7-tetracarboxylate (3) :

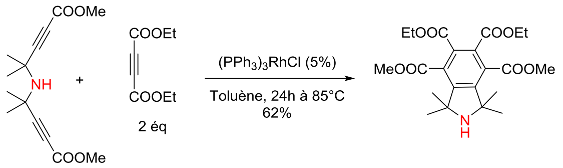

2 3

***Scheme 6** : Synthesis of 5,6-diethyl 4,7-dimethyl 1,1,3,3-tetramethylisoindoline-4,5,6,7-tetracarboxylate (5b).*

In a 250 mL three-necked flask equipped with a magnetic stirrer and a condenser, 0.17 g (0.18 mmol) of tris(triphenylphosphine)rhodium(I) were dissolved in 20 mL dry toluene, and the solution was degassed by argon bubbling. Using a syringe, a degassed solution containing 1.00 g (3.77 mmol) of dimethyl 4,4'-azanediylbis(4-methylpent-2-ynoate) and 1.35 g (7.54 mmol) of diethyl but-2-ynedioate in 1 mL toluene was added dropwise. The reaction mixture was stirred at 85 °C under an argon atmosphere for 24 hours. The solvent was removed under reduced pressure, and the crude residue was purified by chromatography (system: SiO_2, Pentane/AcOEt 1:1), affording 0.99 g of a brown solid in 62% yield.

^{1}H NMR (300 MHz, $CDCl_3$) : **δ** (ppm): 4.32 (q, J= 7.2 Hz, 4H), 3.81 (s, 6H), 1.48 (s, 12H), 1.26 (t, J= 7.2 Hz, 6H).

^{1}H NMR (75 MHz, $CDCl_3$) : **δ** (ppm) : 166.84 (C), 165.96 (C), 131.55 (C), 130.05 (C), 62.3 (CH_2), 52.65 (CH_3), 30.98(C_q), 29.97 (CH_3), 13.92 (CH_3).

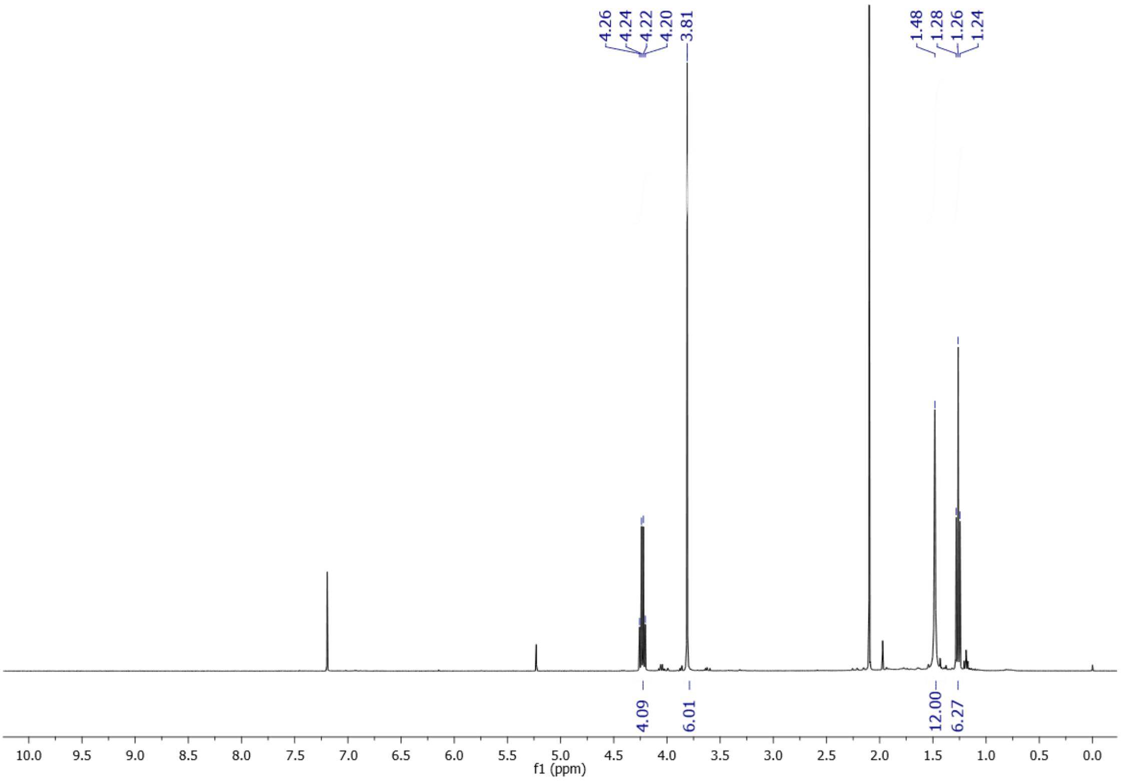

Figure S12: *^{1}H NMR Spectrum (400 MHz, $CDCl_3$) of 5,6-diethyl 4,7-dimethyl 1,1,3,3-tetraméthylisoindoline-4,5,6,7-tetracarboxylate (**3**).*

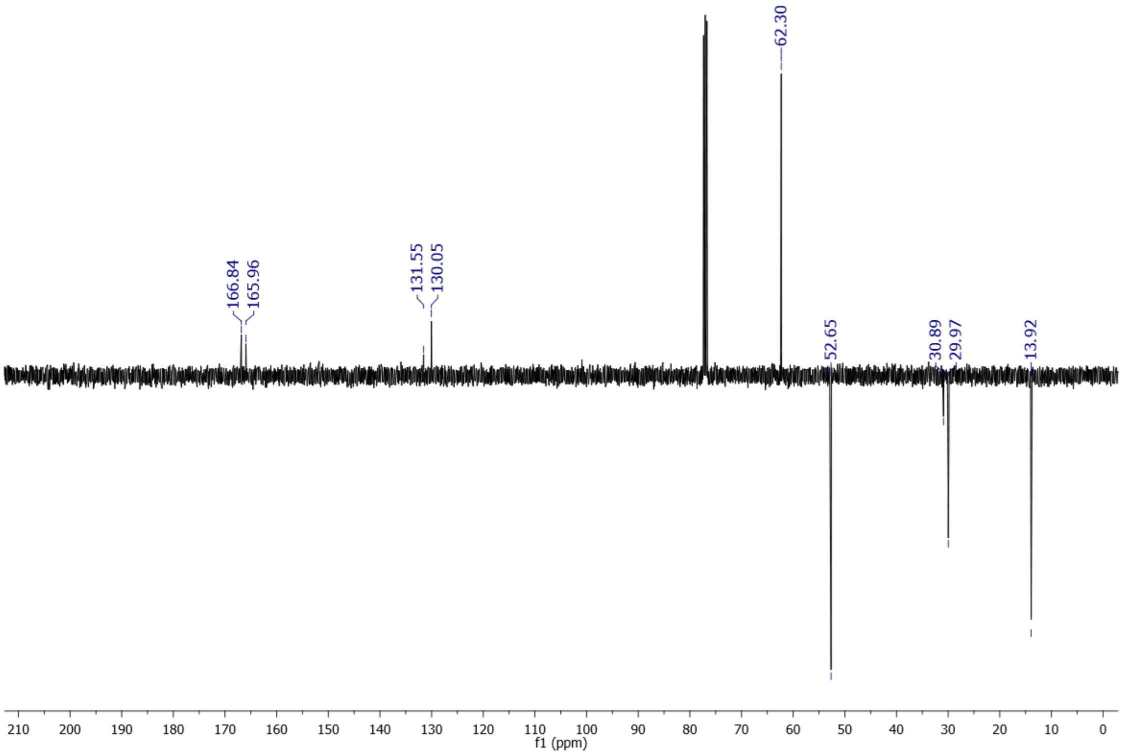

Figure S13: *^{13}C – APT NMR spectrum (101 MHz, CDCl$_3$) of 5,6-diethyl 4,7-dimethyl 1,1,3,3-tetramethylisoindoline-4,5,6,7-tetracarboxylate (**3**).*

Synthesis of 5,6-diethyl 4,7-dimethyl 2-oxyl-1,1,3,3-tetramethylisoindoline-4,5,6,7-tetracarboxylate (7) :

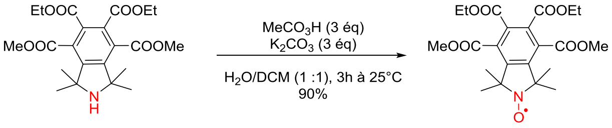

3 7

Scheme 7 *: Synthesis of 5,6-diethyl 4,7-dimethyl 2-oxyl-1,1,3,3-tetramethylisoindoline-4,5,6,7-tetracarboxylate (**7**).*

A solution of 1,1,3,3-tetramethylisoindoline-4,5,6,7-tetracarboxylate (**3**, 0.90 g, 2.07 mmol) in CH_2Cl_2 (50 mL) and H_2O (50 mL) was placed in a 250 mL three-necked round-bottom flask equipped with a magnetic stir bar. A 35% solution of peracetic acid in AcOH (0.89 g, 4.13 mmol) and an aqueous solution of K_2CO_3 (35%, 1.63 g, 4.13 mmol) were added dropwise under vigorous stirring. The mixture was stirred at 25 °C for 3 h. The layers were separated, and the aqueous phase was extracted with CH_2Cl_2 (3 × 100 mL). The combined organic layers were dried over Na_2SO_4, filtered, and concentrated under reduced pressure. The crude residue was purified by column chromatography (SiO2, pentane/EtOAc) to afford the desired compound as a yellow solid (0.84 g, 90%).
ESI-MS: calcd for \[M+Na]+ 450.1764Da; found 450.1764 Da.

EPR (9 GHz, 1 mM in MeOH, 20 °C): aN = 1.45 mT, 3 lines.

Synthesis of 1,1,3,3-tetramethylisoindoline2-oxyl-4,5,6,7-tetracarboxylate de sodium (TC-TMIO) :

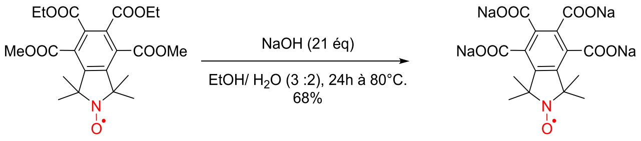

7 TC-TMIO

***Scheme 8** : Synthesis of 1,1,3,3-tetramethylisoindoline2-oxyl-4,5,6,7-tetracarboxylate de sodium (**TC-TMIO**).*

A solution of 1,1,3,3-tetramethylisoindoline-2-oxyl-4,5,6,7-tetracarboxylate diethyl dimethyl ester (7,0.84 g, 1.86 mmol) in H_2O (20 mL) and EtOH (30 mL) was placed in a 250 mL three-necked round-bottom flask equipped with a magnetic stir bar and a reflux condenser. Solid NaOH (1.57 g, 39.16 mmol) was added portionwise, and the mixture was stirred at 80 °C for 24 h. The reaction mixture was concentrated under reduced pressure, and the aqueous phase was adjusted to pH 7 using 10% aqueous HCl. The solution was washed with CH_2Cl_2 (2 × 100 mL). The aqueous phase was further acidified to pH 1–2 with 10% aqueous HCl and extracted with CH_2Cl_2 (3 × 150 mL). The combined organic layers were dried over Na_2SO_4, filtered, and concentrated under reduced pressure. The resulting residue (0.56 g) was dissolved in H_2O (10 mL), NaOH (0.24 g, 6.00 mmol) was added, and the mixture was stirred at 25 °C for 1 h. Lyophilization afforded the target compound as a yellow solid (0.58 g, 68%).

1H NMR (300 MHz, D_2O): the radical was reduced by addition of ascorbic acid prior to measurement. δ = 1.54 (s, 12H).

13C NMR (75 MHz, D_2O): the radical was reduced by addition of ascorbic acid prior to measurement. δ = 174.9 (C), 174.61 (C), 135.36 (C), 135.08 (C), 133.3 (C), 71.83 (C), 23.52 (CH3).

ESI-MS: calcd for \[M+Na]+ 476.9995 Da; found 477.0001 Da.

EPR (9 GHz, 1 mM in H2O, 20 °C): aN = 1.59 mT, 3 lines.

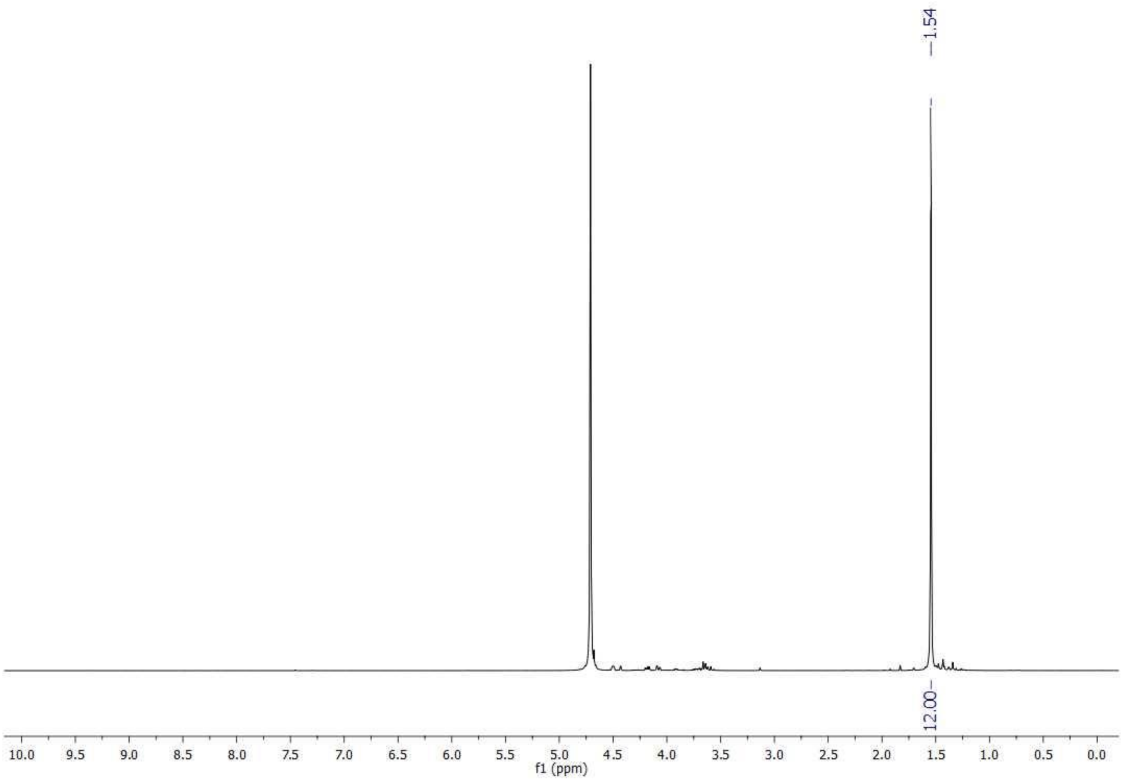

Figure S14: *^{1}H NMR spectrum (300 MHz, D_2O) of 1,1,3,3-tetramethylisoindoline2-oxyl-4,5,6,7-tetracarboxylate de sodium (**TC-TMIO**).*

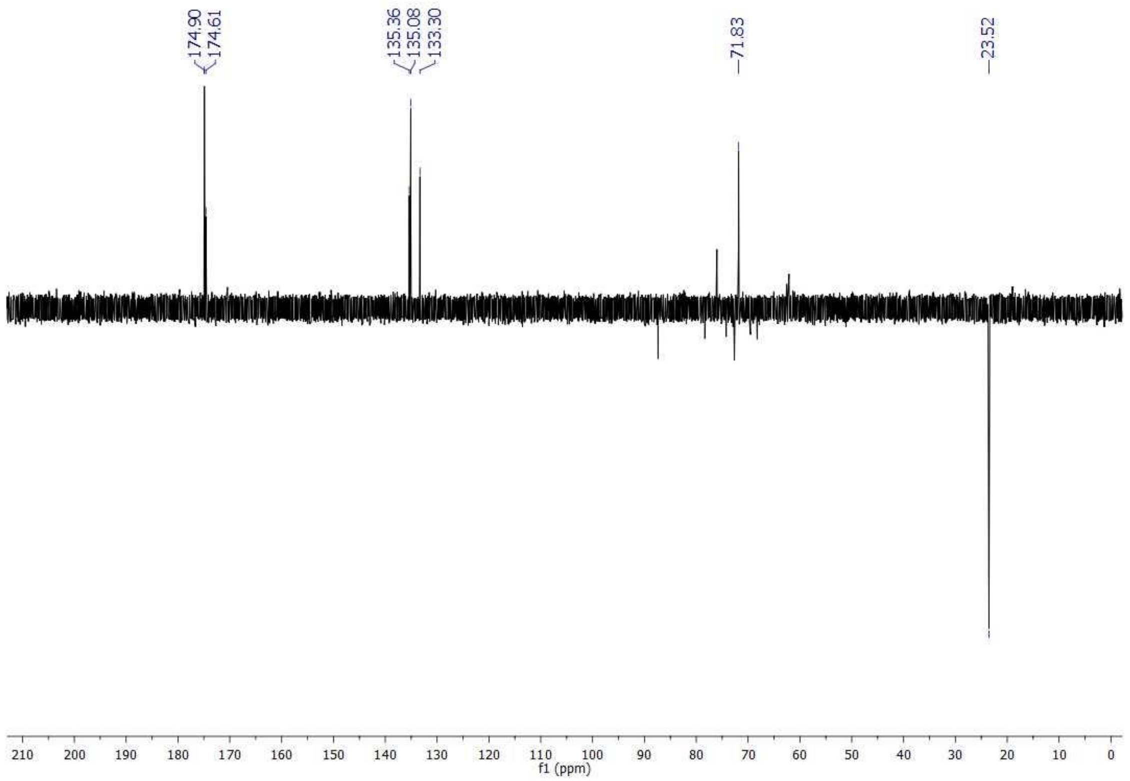

Figure S15: *^{13}C - APT NMR spectrum (75 MHz, D_2O) of 1,1,3,3-tetramethylisoindoline2-oxyl-4,5,6,7-tetracarboxylate de sodium (**TC-TMIO**).*

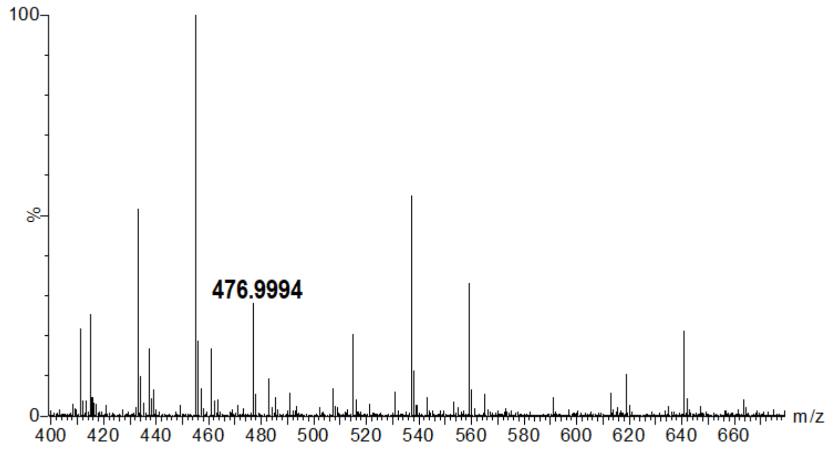

Figure S16: *High resolution mass spectrum (ESI$^+$) of 1,1,3,3-tetramethylisoindoline2-oxyl-4,5,6,7-tetracarboxylate de sodium (**TC-TMIO**). Expected ion at m/z 476,9994.*

Electrochemical data

From cyclic voltammetry: Diffusion coefficients were determined using the Randles Sevick equation assuming a reversible system. This equation describes the evolution of the peak intensity (Ip) as a function of the scan rate as follows:

$$I_p = 0.446\left(\frac{F^3}{RT}\right)^{1/2} n^{3/2} A D_0^{1/2} C_0^{*} \nu^{1/2}$$

Experimentally, peak current is reported with respect to the square root of the scan rate leading to a linear plot. The slope is then used to determine D assuming that the number of electron transfer is one and the temperature 25°C.

Kinetic rate constant K^0_{app} was determined using the well-known Nicholson's method. This simple method uses the peak potential difference (ΔEp = Epa – Epc) at different scan rate. ΔEp is a function of Ψ, the Nicholson parameter (dimensionless) which is plotted as a function of the inverse of the square root of the scan rate. The slope gives then direct access to K^{0app}.

$$\Psi = \frac{-0.6288 + 0.0021 n\Delta E_p}{1 - 0.017 n\Delta E_p}$$

$$\Psi = K^0 \left(\frac{\pi D n F}{RT}\right)^{-1/2} \nu^{-1/2}$$

$$K^0 = slope\left(\frac{\pi D n F}{RT}\right)^{1/2}$$

From RDE: Diffusion coefficients are obtained from Levich equation that describe the plateau current (I_l) as a function of several parameter including the rotation speed of the electrode (ω).

$$I_l = 0.62 n F A D_0^{2/3} \omega^{1/2} \nu^{-1/6} C_0^{*}$$

Linear sweep voltammetries are recorded at different rotation speeds and I_l is plotted versus the square root of the rotation speed. Assuming that all other parameters are known, the slope of this linear regression gives direct access to the diffusion coefficient D.

Kinetic rate constant is determined form the Koutecky Levich method. Experimentally, linear sweep voltammetries are still recorded at different rotation speed. Then the analysis can be summarized as follow: For several overpotential (η) in the kinetic controlled region, the inverse of I_k is plotted as a function of the inverse of the square root of the rotation speed. The intercept of this graph gives $1/I_{k(\eta)}$. In a second step, a graph η = f(Log I_k) is plotted allowing the determination of I_0 the exchange current

for the electrochemical process. I_0 is the extrapolation of the linear fit of the data at $\eta = 0$. K0 is then deduce from I_0 using the following equation:

$$K^0 = \frac{I_0}{nFAC}$$

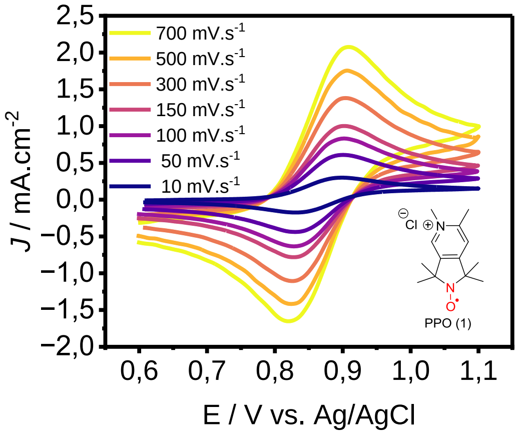

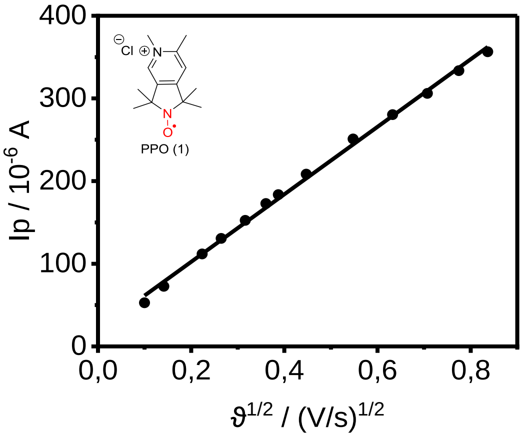

c)

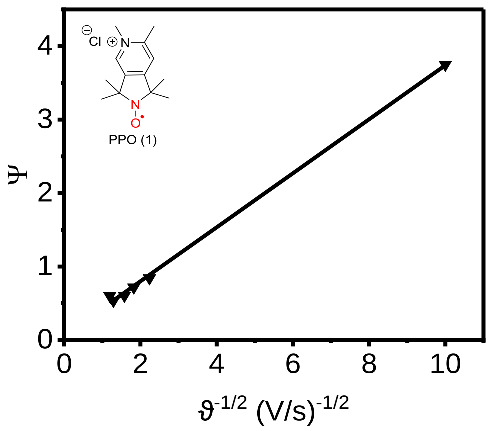

Figure S17: (a) Cyclic voltammograms of PPO (1) recorded at various scan rates (10–700 $mV\cdot s^{-1}$). (b) Plot of the anodic peak current (Ip,ox) versus the square root of the scan rate ($\nu^{1/2}$). (c) Plot of ψ as a function of $\nu^{-1/2}$. Conditions: 5 mM analyte in 1 M NaCl supporting electrolyte; working electrode: glassy carbon (area = 0.0706 cm^2); counter electrode: platinum wire; reference electrode: Ag/AgCl (saturated KCl).

a) b)

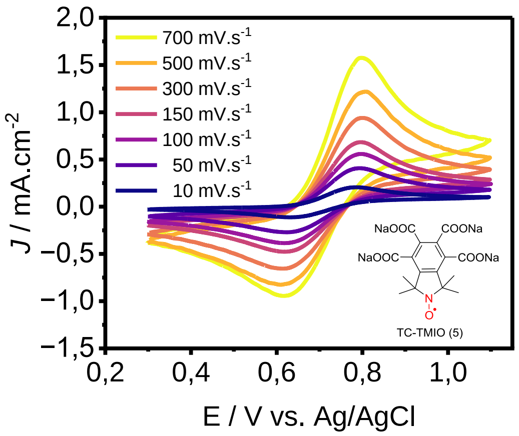

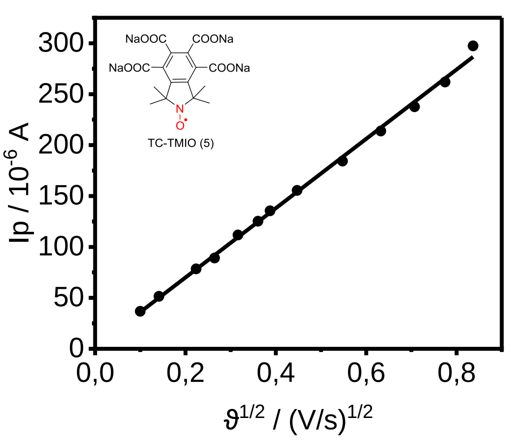

c)

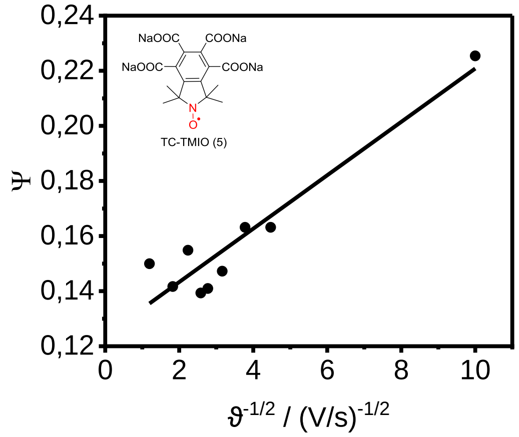

Figure S18: (a) Cyclic voltammograms of TC-TMIO recorded at various scan rates (10–700 mV·s^{-1}). (b) Plot of the anodic peak current (Ip,ox) obtained from CV as a function of the square root of the scan rate ($\nu^{1/2}$). (c) Plot of ψ versus $\nu^{-1/2}$. Conditions: 5 mM analyte in 1 M NaCl supporting electrolyte; working electrode (WE): glassy carbon (area = 0.0706 cm^2); counter electrode (CE): platinum; reference electrode (REF): Ag/AgCl (saturated KCl).

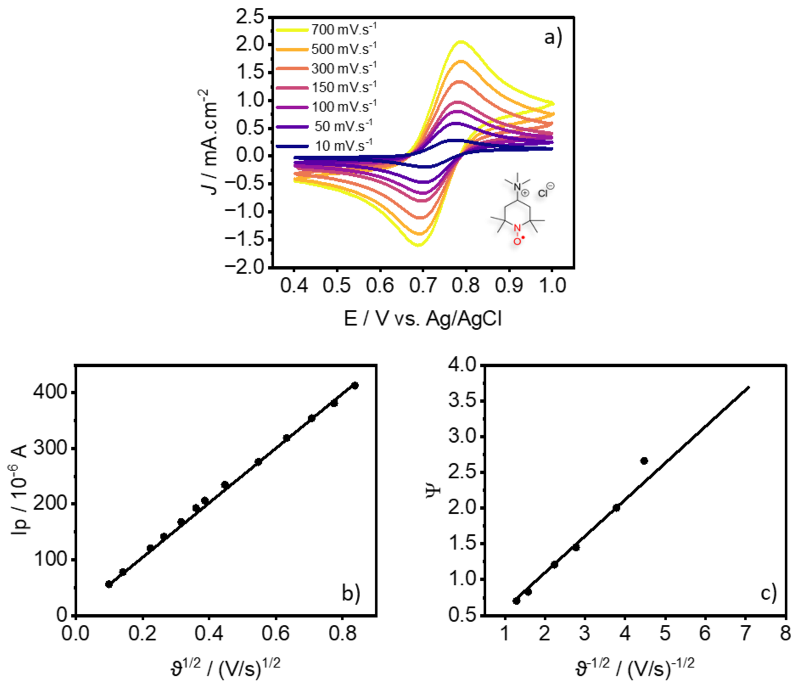

Figure S19: a) Cyclic voltammograms of **4-TMA TEMPO** at various scan rates (10 to 700 mV s^{-1}). b) Plot of I*p* over the square root of scan rates for **4-TMA TEMPO** at various scan rates (10 to 700 mV s^{-1}). c) Plot of ψ over $\vartheta^{-1/2}$ for **4-TMA TEMPO** at various scan rates (10 to 700 mV s^{-1}). Conditions: 5 mM analyte in 1 M NaCl (aq) electrolyte; glassy carbon working electrode; platinum counter electrode; Ag/AgCl reference electrode.

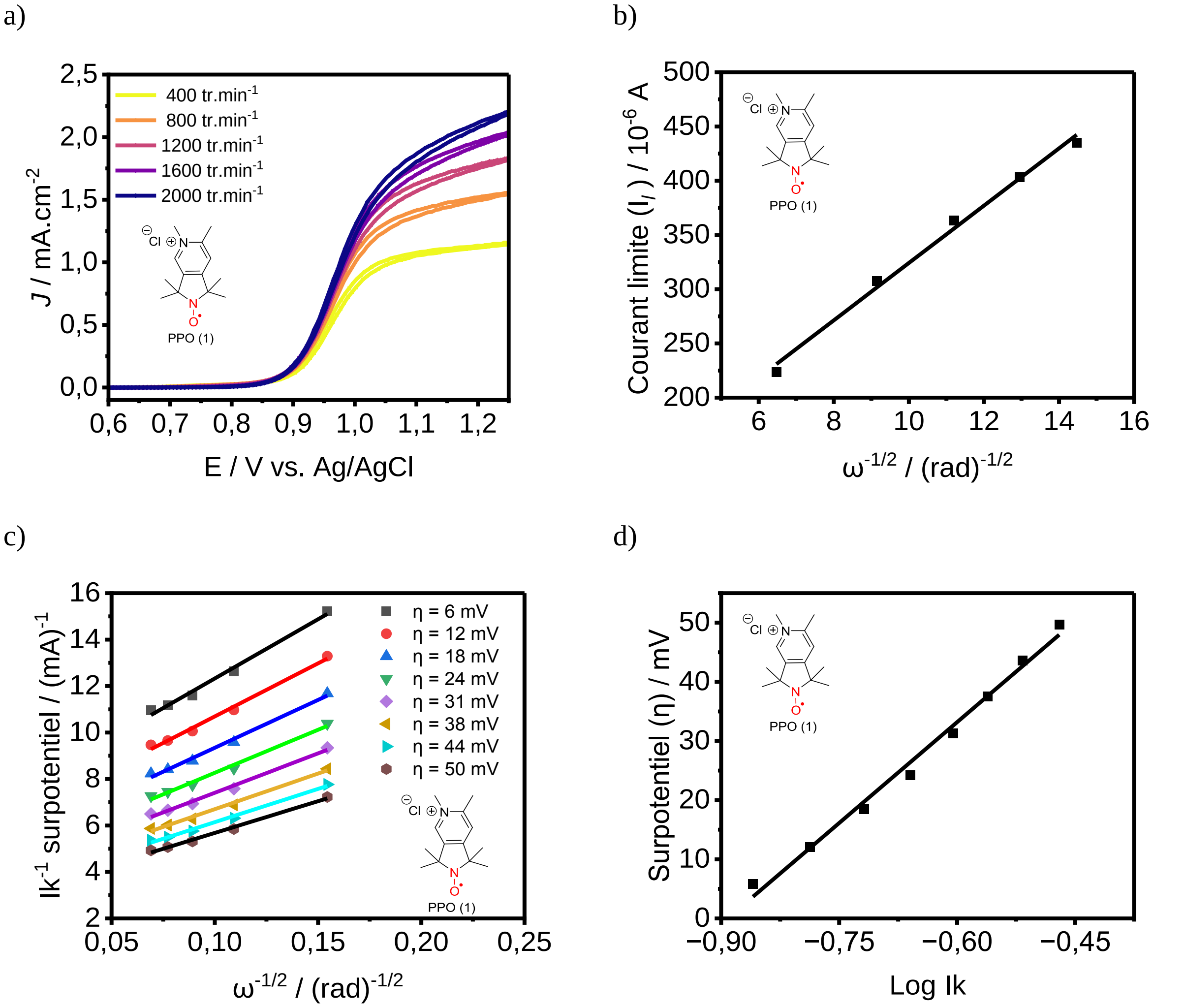

Figure S20: (a) Rotating disk electrode (RDE) voltammograms of PPO (1) recorded at various rotation rates (400–2000 rpm). (b) Plot of I_{la} (measured at 0.99 V) versus the square root of the rotation rate ($\omega^{1/2}$). (c) Koutecky–Levich plots of $I_{K^{-1}}$ versus $\omega^{-1/2}$. (d) Tafel plot of overpotential (η) versus log $I_{K(\eta)}$. Conditions: 5 mM analyte in 1 M NaCl supporting electrolyte; rotating glassy carbon working electrode (area = 0.1963 cm²); platinum counter electrode; Ag/AgCl (saturated KCl) reference electrode.

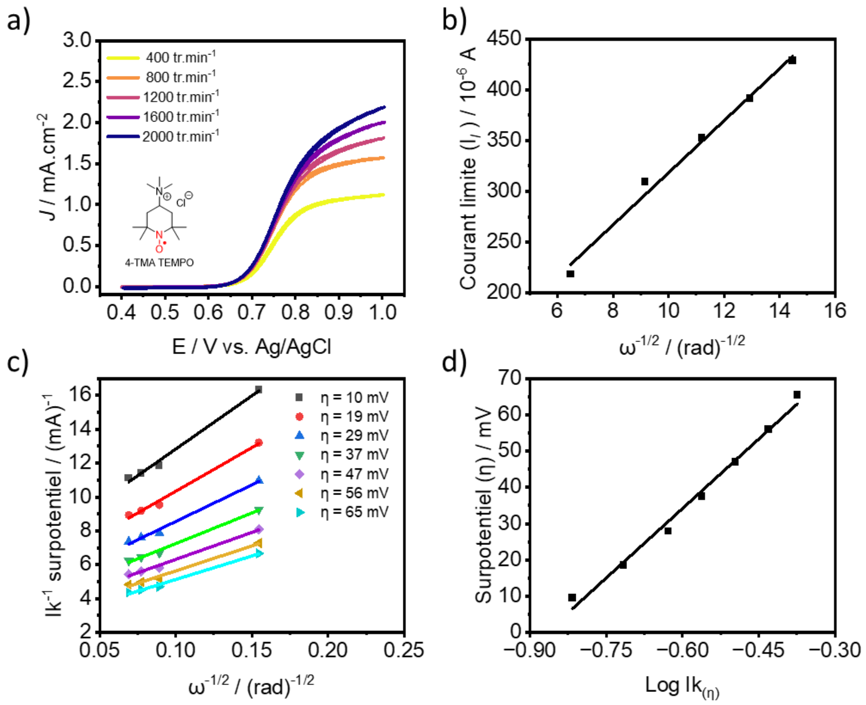

Figure S21: a) Linear sweep voltammograms of **4-TMA TEMPO** at various rotating rates (400 to 2000 rpm); b) Levich plots of the limiting current (measured at 1.25 V) versus the square root of rotation rates for **4-TMA TEMPO**; c) i^{-1} versus $\omega^{-1/2}$ for **4-TMA TEMPO**; d) Overpotential versus the logarithm of kinetic current and the corresponding fitted Tafel plots for **4-TMA TEMPO**. Conditions: 5 mM analyte in 1 M NaCl (aq) electrolyte; working electrode glassy carbon rotating disk electrode; platinum counter electrode; Ag/AgCl reference electrode.

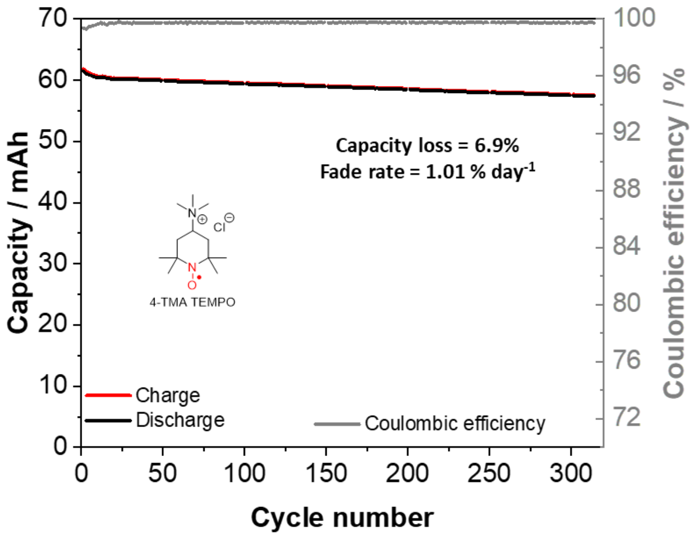

Figure S22: Charge/discharge capacity vs cycle number profiles for MV/**4TMA TEMPO** batteries presented in figure 2. Duration: 6.8 days.

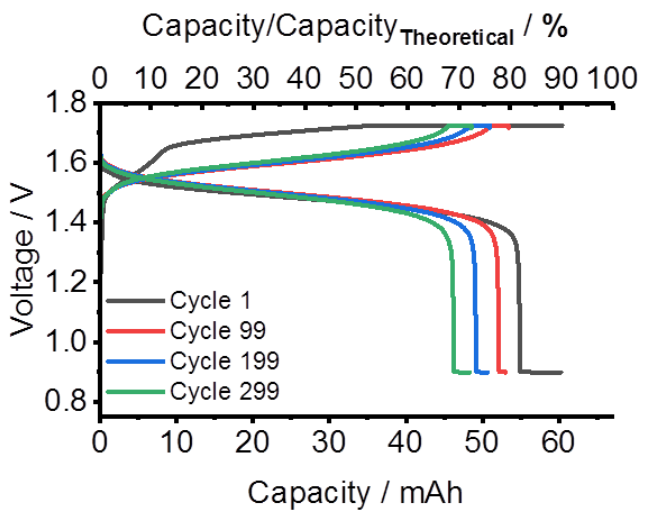

Figure S23: Charge/discharge capacity curves as a function of voltage of **PPO**/**MV** flow battery. Conditions: anolyte: 75 ml of 0.1 M **MV** in 1 M NaCl (aq); catholyte: 25 ml of 0.1 M **PPO** in 1M NaCl (aq); Fumasep FAA-3-50 anion-exchange membrane; current density 10 mA cm^{-2}; temperature 23°C, time 6.8 days

4-TMA TEMPO / MV flow battery			PPO / MV flow battery		
Cycles	Capa. loss/ Capa. max (%/ cycle)	Capa. loss/ Capacity max (%/ day)	Cycles	Capa. loss/ Capa. max (%/ cycle)	Capa. loss/ Capacity max (%/ day)
50 to 100	0,01624	0,74	50 to 100	0,0474	2,28
100 to 150	0,01448	0,66	100 to 150	0,0440	2,14
150 to 200	0,01520	0,70	150 to 200	0,0401	1,95
200 to 250	0,01740	0,81	200 to 250	0,0409	1,98
250 to 300	0,01499	0,70	250 to 300	0,0420	2,03

Table S1. Experimentally derived average capacity fade rates using linear regression for both 4-TMA TEMPO / MV flow battery experiment and the PPO / MV flow battery experiment.

Polarization curves for the MV/PPO battery :

a) b)

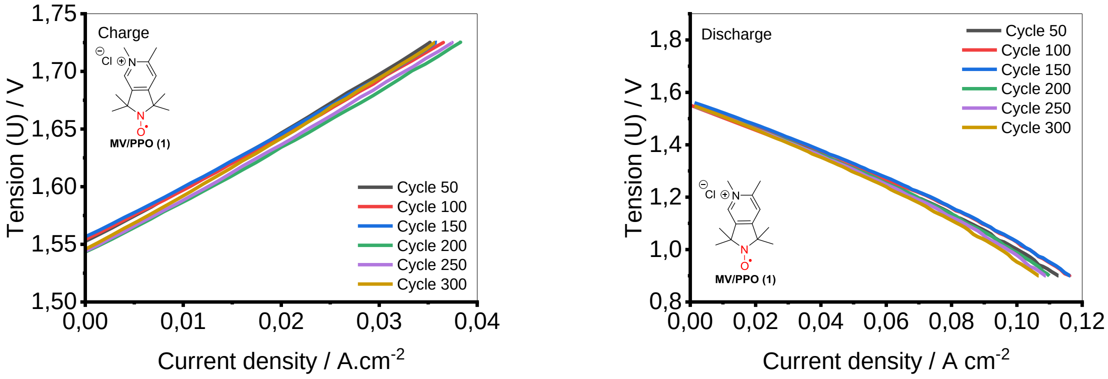

Figure S24: Polarization curves at 50% SOC of the MV/PPO redox flow battery: (a) charge; (b) discharge.

Polarization conditions: 20 A·min⁻¹; anolyte: 75 mL of 0.1 M MV in 1 M NaCl (aq); catholyte: 25 mL of 0.1 M PPO in 1 M NaCl; anion-exchange membrane: Fumasep FAA-3-50; temperature: 23 °C; total operation time: 6.8 days.

a) Negolyte b) Posolyte

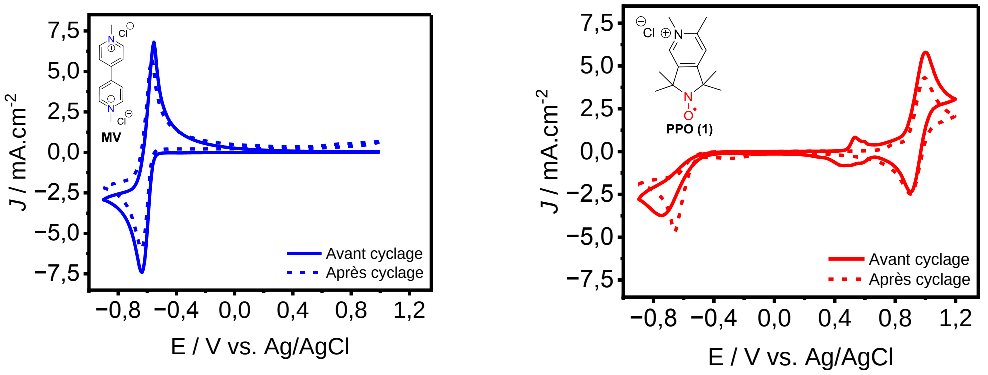

Figure S25: Cyclic voltammograms of the MV (a) and PPO (b) electrolytes before and after cycling.

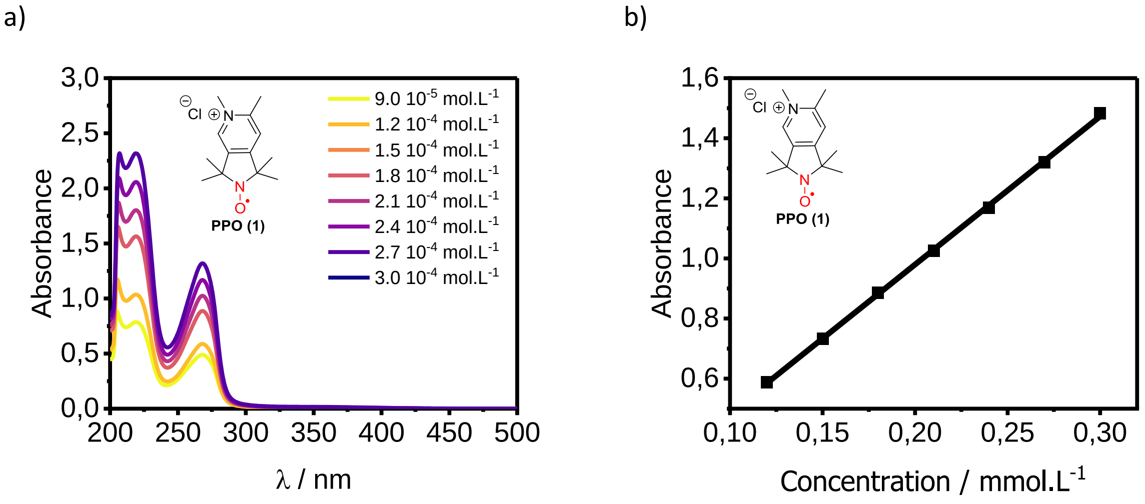

Figure S26: (a) UV–vis spectra of PPO (1) at various concentrations. (b) Calibration curve of PPO (1).

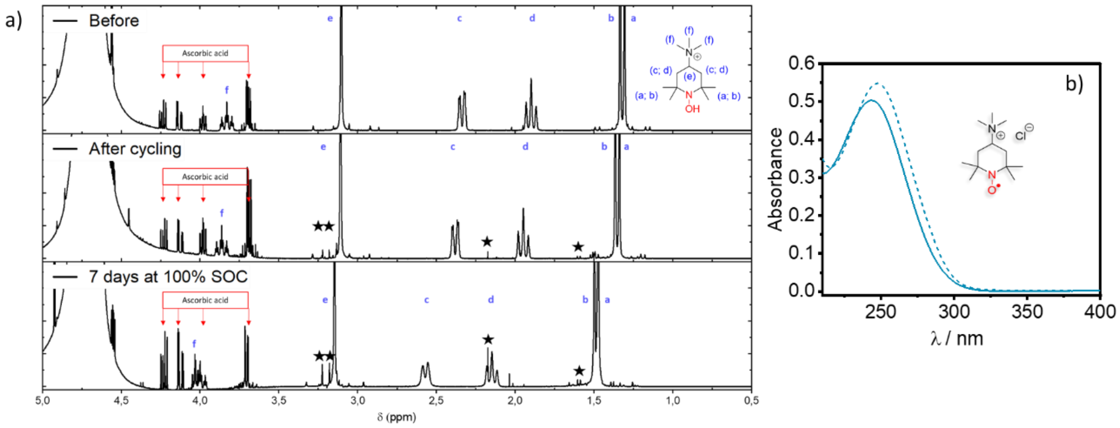

Figure S27: a) ^{1}H NMR spectra of the 4-TMA TEMPO electrolyte before and after cycling, and after being stored for 6.8 days in the oxidized state (100% SOC). b) UV-visible absorbance spectra of posolytes containing 4-TMA TEMPO before(plain) and after (dashed) cycling.
